# Inhibitory control development from infancy to early childhood: A longitudinal fNIRS study

**DOI:** 10.1016/j.dcn.2025.101557

**Published:** 2025-03-26

**Authors:** Abigail Fiske, Alicia Mortimer, Liam Collins-Jones, Carina C J M de Klerk, Sylvia Ulieta Gattas, Henrik Dvergsdal, Gaia Scerif, Karla Holmboe

**Affiliations:** a Department of Experimental Psychology, Medical Sciences Division, University of Oxford, Oxford, United Kingdom; b Department of Psychology, Lancaster University, Lancaster, United Kingdom; c Department of Human Development and Quantitative Methodology, University of Maryland, College Park, MD, United States; d Department of Clinical Neurosciences, University of Cambridge, Cambridge, United Kingdom; e Centre for Brain Science, Department of Psychology, University of Essex, Colchester, United Kingdom; f Department of Psychology and Human Development, Institute of Education, Faculty of Education and Society, University College London, London, United Kingdom; g School of Psychology, Faculty of Health and Medical Sciences, University of Surrey, United Kingdom; h Nord University Business School, Department of Entrepreneurship, Innovation and Organisation, Bodø, Norway; i School of Psychological Science, University of Bristol, Bristol, United Kingdom

**Keywords:** Executive function, Response inhibition, Functional near-infrared spectroscopy, Prefrontal cortex, Parietal cortex

## Abstract

The developmental period from infancy to early childhood is one of substantial change – in advancements in cognitive skills, such as early executive functions, but also in the maturation of the prefrontal and parietal cortices that parallel such advances. The current study aims to investigate the emergence and development of inhibitory control, a core executive function, from infancy to early childhood. We collected longitudinal functional near-infrared spectroscopy (fNIRS) data from the same sample of participants at 10-months, 16-months, and 3½ years of age whilst they completed the *Early Childhood Inhibitory Touchscreen Task*. In our previous publications, we reported that 10-month-old infants recruited right lateralised regions of the prefrontal and parietal cortex when inhibition was required. Despite no change in response inhibition performance, 16-month-olds recruited broader and bilateral regions of the prefrontal and parietal cortex. Results of the current study found that 3½-year-olds activated regions of the right inferior parietal cortex and the right inferior frontal gyrus when inhibition was required. Response inhibition performance was significantly improved by early childhood, yet there was commonality in the brain regions recruited at 16-months and 3½ years. This could suggest that these brain regions are fundamental neural indices of inhibitory control, even from toddlerhood.

## Highlights

• The ECITT is a valid measure of response inhibition from 10 months to 3½ years.
• No response inhibition change from 10 to 16 months, but large improvement by 3½ years.
• Right inferior parietal and right inferior frontal cortex recruited during response inhibition across ages.

## 1 Introduction

The early years of life are abundant with important milestones. Underscoring this rich developmental period is the emergence and development of executive functions (EFs); fundamental cognitive skills that serve to guide goal-directed behaviours (Diamond, 2013). Knowledge about early EFs is growing quickly (for review, see Morgan et al., 2025), and it is now clear that basic forms of these cognitive skills emerge already in the first year of life (Cuevas and Bell, 2014, Hendry et al., 2021, Holmboe et al., 2008). Early EF skills develop rapidly across the first three years (Hendry et al., 2016) and there is increasing evidence to suggest that this development is paralleled by significant maturation of the prefrontal and parietal cortices (Diamond, 2002, Fiske and Holmboe, 2019). However, knowledge is limited about how functional changes in the cortex may support the development of EF skills across the early years of life. By examining neural development in early life, it may be possible to identify and understand potential mechanisms that relate to cognitive development at an earlier age than is possible with behavioural studies. The current pre-registered study uses longitudinal behavioural and neuroimaging data to investigate the development of inhibitory control, a core executive function, from its emergence in infancy and across the first 3½ years of life.

### 1.1 The development of early executive functions

Commonly within the literature, component EF skills such as working memory, cognitive flexibility and inhibitory control are measured in infancy and early childhood (Johansson et al., 2016, Mulder et al., 2014, Wiebe et al., 2008). This current study focuses specifically on the development of *response inhibition*, a type of inhibitory control that enables us to stop (inhibit) a response that is automatic, habitual or preferred (prepotent). Response inhibition can be measured already in infancy (Holmboe et al., 2018), and improves significantly across early childhood (Kochanska et al., 2000, Wiebe et al., 2012). Toddlerhood appears to be an important transitional period for the development of early EF skills, as evidence suggests that individual differences in early EF begin to stabilise from around 24-months (Broomell and Bell, 2022, Carlson et al., 2004, Holmboe et al., 2021, Hughes and Ensor, 2007). It is argued by Clark et al. (2016) that fundamental changes in children’s ability to exert control occur during early childhood, as evidenced by age-related improvements in different facets of inhibition (assessed with varying task demands) that occur simultaneously in the preschool period. Some suggest that this improvement in EF performance represents a shift in modes of control employed by young children, from a reactive to a more proactive exertion of control, such that control is exerted in anticipation of events (instead of in response to events) (Chatham et al., 2009, Chevalier et al., 2015, Clark et al., 2016, Waxer and Morton, 2011).

Overall, evidence points to the development of more sophisticated and integrated EF skills across the early childhood period (Howard et al., 2015). However, much of the evidence comes from cross-sectional studies. Whilst these studies highlight snapshot differences in EF performance between age groups, it is not possible to examine true developmental *change* in EF skills without longitudinal research. Previous longitudinal studies have tracked the development of early EF skills, pointing to the improvement of early EFs across early childhood (Broomell and Bell, 2022, Devine et al., 2019, Holmboe et al., 2018, Johansson et al., 2016). However, this research has been limited by the availability of age-appropriate tasks that reliably measure early EF skills in infancy but that can also be used across toddlerhood and into early childhood (Holmboe et al., 2021). Whilst changing tasks is often unavoidable, conclusions drawn about change over time are limited. This is because it cannot be determined whether change is driven by age-related improvement, or rather by changes in task design that result in a fundamentally different construct being measured. As such, there is a clear need for longitudinal research that investigates early EF skills from infancy to early childhood using the *same task* to provide a clear picture of the development of these advanced cognitive functions.

### 1.2 The neural correlates of early executive functions

For decades, research has drawn parallels between the development of the prefrontal cortex (PFC) and improvements in cognitive abilities observed in childhood and adolescence (Bathelt et al., 2017, Bathelt et al., 2018, Fiske and Holmboe, 2019, Gibson, 1991, Gogtay et al., 2004, Goldman-Rakic, 1987, Romine and Reynolds, 2005). Using child-friendly neuroimaging techniques such as electroencephalography (EEG), research has shown clear links between the PFC and early EF skills already in infancy (Bell et al., 2007, Bell and Fox, 1992, Cuevas et al., 2012). More recently, studies using functional near-infrared spectroscopy (fNIRS) have highlighted the role of the prefrontal and parietal cortices during EF tasks with infant and toddler participants (Fiske et al., 2022, Fiske et al., 2024, Kerr-German et al., 2022, Reyes et al., 2020). However, there is a gap in the literature whereby very little is known about the neural correlates that might support the *developmental trajectory* of early EF skills across infancy and early childhood.

Research is hindered by the lack of age-appropriate tasks for measuring early EF (as previously discussed), but also by the difficulty in collecting neuroimaging data from young populations (Margolis et al., 2025). However, we can begin to piece together a (fragmented) developmental trajectory of inhibitory control based on research with older children and adults. This research has pointed to regions of the PFC, particularly the right inferior frontal gyrus (IFG), and regions of the parietal cortex, as key neural substrates of response inhibition (Aron et al., 2014, Cope et al., 2020, Hampshire et al., 2010, Kolodny et al., 2017). In contrast to the adult findings, the role of the left hemisphere, particularly the left dorsolateral PFC (DLPFC), has been shown to play an important role in supporting inhibitory control performance in 3- to 5-year-olds (Li et al., 2017). There is also evidence that suggests that the continued maturation of the bilateral frontal-parietal brain network is fundamental for the development of improved response inhibition performance from 4- to 6 years (Mehnert et al., 2013). Similarly, in slightly older children (6 – 10 years), fMRI research by Durston et al. (2002) found increased activation in the bilateral OFC, the right parietal cortex and the right DLPFC during inhibitory demanding trials but importantly noted that activation in these areas was greater for children than for adults.

Although neuroimaging research in toddlerhood is limited, some fNIRS evidence points to the involvement of fronto-parietal networks in the left hemisphere and bilateral parietal networks in supporting successful inhibition in 2½ year olds (Kerr-German et al., 2022). Similarly, our own fNIRS research has highlighted the consistent recruitment of the prefrontal and parietal cortices when inhibition is required across the transition from infancy to toddlerhood (Fiske et al., 2022, Fiske et al., 2024). We found that 10-month-old infants recruited right-lateralised regions of the PFC and parietal cortex when inhibition was required, but by 16-months, the same participants recruited the left superior parietal gyrus, the right inferior frontal gyrus, and bilateral regions of the dorsolateral PFC and orbital frontal cortex (Fiske et al., 2022, Fiske et al., 2024). Although there was no longitudinal change in response inhibition performance, more widespread, bilateral regions of the PFC were recruited during response inhibition at 16-months compared to 10 months (Fiske et al., 2024). We interpreted this to suggest that the transition from late infancy into toddlerhood represents a fundamental period of reorganisation in the PFC that then functions as a scaffold to support the improvement of behavioural response inhibition into early childhood.

An important question of interest for the current study is whether prefrontal and parietal activation during inhibitory control tasks in early childhood will be right-lateralised (as is often found in adult studies of inhibitory control, e.g., Aron et al. 2014 and in infancy (Fiske et al., 2022)), or whether activation will be found in both hemispheres, as is more typical in studies with toddlers (Fiske et al., 2024, Kerr-German et al., 2022). Existing research with children (aged 3 – 10 years) has found evidence for bilateral prefrontal and parietal activation (Berger et al., 2022, Eng et al., 2022, Li et al., 2017) but evidence also exists for continued right-lateralisation when inhibition is required (Mehnert et al., 2013, Zhou et al., 2022). However, at present, the relationship between brain and behaviour when examining early EF development is not yet fully understood. It may be that the current state of the brain creates the foundation for cognitive growth, or that cognitive development is the driving force behind neural reorganisation (Kievit and Simpson-Kent, 2019). As such, longitudinal studies may offer the key to understanding the complex and dynamic interactions between cognitive development and neural maturation over time (Kievit and Simpson-Kent, 2019).

Overall, there remain several unknowns in our understanding of early inhibitory control development – both at the level of cognitive performance and the neural correlates. The aim of the current longitudinal study is to better understand the mechanisms through which neural processes and cognition interact as inhibitory control skills emerge and develop early in life.

### 1.3 The current study

The current pre-registered study aims to identify the brain regions that support response inhibition in early childhood (3½ years), and to longitudinally examine developmental change in both behavioural and neural indices of response inhibition from infancy to early childhood. Whilst previous longitudinal research has examined resting state functional connectivity development in infancy (Damaraju et al., 2014), as well as the functional activation underpinning working memory in infancy (Reyes et al., 2020) and cognitive flexibility in early childhood (Moriguchi and Hiraki, 2011), no previous longitudinal fNIRS studies have investigated the behavioural development and neural correlates of inhibitory control from infancy to early childhood. As such, this current study aims to present a developmental trajectory of response inhibition across the first 3½ years of life.

## 2 Method

### 2.1 The Oxford Early Executive Functions Study

The data presented in this current study were collected as part of “The Oxford Early Executive Functions (OEEF) Study”, or pilot test sessions for this study, which was led by Dr Karla Holmboe at the University of Oxford. The OEEF study is a comprehensive longitudinal study of the development of early EFs. Participants were recruited via the Oxford University Babylab database, where families with young children were sent an email inviting them to participate. Parents / caregivers completed a background questionnaire, which included questionnaires designed to assess early Autism traits, and were asked to report whether there was a close family history of Autism or ADHD. However, no clinical screening for developmental delays took place, and no clinical diagnoses were disclosed. As such, we consider the OEEF sample to be a typically developing sample.

Assessment points were planned at 10, 16, 24 and 30 months of age. However, severe disruption due to the COVID-19 pandemic meant that testing needed to be paused for 1½ years as a result of repeated laboratory closures. This caused significant attrition at the 16-month assessment point (only 45 % of participants had their 16-month sessions) and meant it was not possible to collect data at 24 and 30 months. An additional assessment point was added when children were 42 months (3½ years) which ran from August 2021 until November 2022. Participants visited the laboratory for two testing sessions (spaced about a week apart) at 10, 16 and 42 months of age.

Publications exist that have used a sub-set of the OEEF data to examine inhibitory control development in infancy (Lui et al., 2021), across the transition to toddlerhood (Hendry et al., 2021) and at pre-school age (Mortimer et al., 2024). Additionally, our previous research has used fNIRS to examine the neural correlates of early inhibitory control development when the cohort were 10-months (Fiske et al., 2022) and 16-months of age (Fiske et al., 2024). None of these previous publications have reported on the full trajectory of inhibitory control development at the behavioural and neural levels between 10 months and 3½ years of age.

Across our previous publications, the ECITT (Holmboe et al., 2021) was used to measure response inhibition (the ability to inhibit a prepotent response). This task can be used from as young as 9–10-months (Hendry et al., 2021, Rico‐Picó et al., 2025), across toddlerhood (18- to 24-months), and with minor modification, across the lifespan (Holmboe et al., 2021). The current study uses the ECITT to longitudinally examine (in the same cohort of children) change in response inhibition performance from infancy, across the transition to toddlerhood, and into early childhood. With this, we aim to provide, for the first time, a developmental trajectory of response inhibition development across the first four years of life, in the same sample of children, using the same task.

Two versions of the ECITT exist and were administered at all assessment points in the OEEF study. In the first testing session, we administered the “behavioural” ECITT (as described in Hendry and Holmboe, 2021; Lui et al., 2021; Mortimer et al., 2024). In the second testing session, the “blocked” ECITT, specifically designed to be administered alongside fNIRS (as described in Fiske et al., 2022, Fiske et al., 2024), was used. The task was identical at 10- and 16-months, but slight modifications were made at 42-months to ensure the task remained age-appropriate (described below). All data presented in the current study were from the “blocked” ECITT.

### 2.2 Participants and exclusions

Participants were 127 3½-year-olds who were recruited to the Oxford Early Executive Functions (OEEF) study (or pilot sessions for this study) shortly before they were 10-months of age (University of Oxford Central University Research Ethics Committee: R57972/RE010). Families were invited to attend two testing sessions at the Oxford University Babylab (∼ 1.5 hours each, including breaks) where children completed a battery of age-appropriate EF tasks. Written informed consent was obtained from the caregivers of all participants before starting the study. Families received a £ 20 online shopping voucher and a Babylab branded gift for their participation.

As per the OEEF study inclusion criteria, one female participant was excluded from the study due to low birth weight, and three male participants were excluded due to birth complications leading to health-related concerns. Following this, 121 children contributed blocked ECITT data, and 100 children contributed fNIRS data to this study. The final sample sizes after data exclusions for all assessment points are reported in Table 1 (for details about exclusions, see Supplementary Materials 1.1). Full demographic information for the sample of 3½-year-old participants is available in Supplementary Materials 1.2.

**Table 1:** Final sample sizes at all assessment points.

Data	Assessment Points	N
Blocked ECITT	10-months	121
	16-months	81
	3½ years	100
	All	38
	10-months and 3½ years	78
	16-months and 3½ years	44
fNIRS	10-months	59
	16-months	43
	3½ years	61
	All	9
	10-months and 3½ years	25
	16-months and 3½ years	16

*Note.* For fNIRS data to be considered valid, participants must also have contributed valid blocked ECITT data.

### 2.3 Apparatus and stimuli

#### 2.3.1 Gowerlabs NTS fNIRS system

**Fig. 1:**
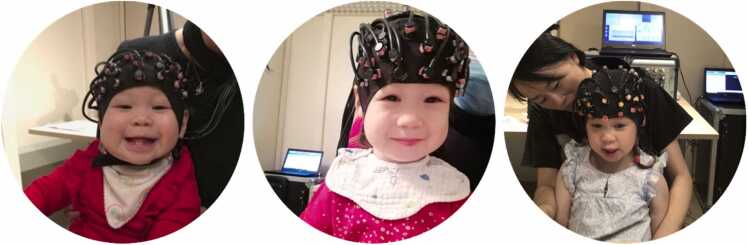
fNIRS Cap Placement. Note. Photographs of the same participant wearing the fNIRS cap at 10-months, 16-months and 3½ years of age. Consent for the use of this image has been obtained from the participant's parent. For more information about cap placement standardisation, please see Supplementary Materials 2.2.

The Gowerlabs NTS continuous wave fNIRS system was used to collect brain data from participants (Fig. 1). The fNIRS probe consisted of 32 optical sensors forming 46 channels that bilaterally covered the PFC and the area around the intraparietal sulcus. The fNIRS system and optode configuration was identical to that used at previous assessment points (Fiske et al., 2022, Fiske et al., 2024). A channel map, sensitivity profile of the fNIRS array (Fiske et al., 2022, Fig. 1) and a visualisation of the fNIRS array projected onto an image of the cortex (Fiske et al., 2024; Fig. 1) can be found in Supplementary Materials 2.1.

#### 2.3.2 Early Childhood Inhibitory Touchscreen Task (ECITT)

The ECITT (Holmboe et al., 2021) was designed specifically to measure response inhibition (i.e., the ability to inhibit a prepotent response) in infants and young children. Participants are presented with an iPad that has two blue “buttons” on either side of the screen – one displays a smiley-face icon (target), the other is blank. On the majority of trials (75 %), the target appears on the same side of the screen (left or right – counterbalanced between participants) and so a prepotent response is developed (“prepotent trials”). The prepotent side remained the same across all testing sessions and assessment points for each participant. On the remaining trials (25 %), the target appears on the opposite side of the screen and participants are required to inhibit their prepotent response and instead make an alternative response to the other side of the screen (“inhibitory trials”). Whilst the task was identical at the 10- and 16-month assessment points, minor modifications were made to ensure age-appropriateness for the 3½-year-old participants (described below). This included a decrease (from ∼4 s to ∼1 s) in the duration of the animations (reward) after each trial. This was done to create a faster pace (and thereby higher difficulty level) and to keep the interest of the older children.

**Fig. 2:**
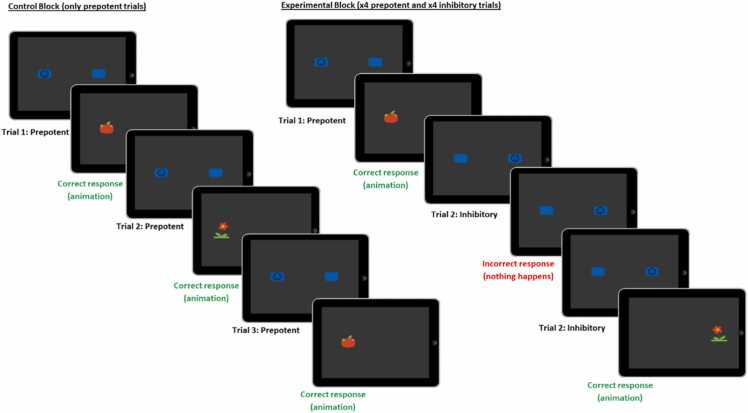
The blocked ECITT.

At 10- and 16-months, the blocked ECITT (Fig. 2) consisted of *control blocks* (six prepotent trials; no inhibitory demand) and *experimental blocks* (three prepotent and three inhibitory trials) that were separated by a *baseline* block (moving abstract shapes accompanied by calm music, jittered in duration from 12 – 17 s). Trials within the *experimental block* were randomly presented with the constraints that the target always appeared on the prepotent side on the first trial and that the target could not appear consecutively in the same location more than twice. The experimenter stopped the task when the infant became fussy or disengaged with the task. At 3½ years, there were eight trials per block (*control block* = eight prepotent trials, *experimental block* = four prepotent, four inhibitory trials). The experimenter stopped the task once the participant had completed at least four blocks of each type. The number of blocks completed by participants with valid fNIRS data at 3½ years ranged from 3 – 6 of each block type (Control: *M* = 4.34, Experimental: *M* = 4.31), with a mean duration of 18.5 s (*SD* = 2.8 s) for control blocks and 19.3 s (*SD* = 2.5 s) for experimental blocks. In total, 3½-year-old participants with valid fNIRS data completed between 6 – 12 blocks of trials (*M* = 8.66), which is equivalent to 48 – 96 individual trials. The code for the ECITT is available on Figshare and the stimuli are available on OSF.

### 2.4 Procedure

The administration procedure was largely identical to that described for both the 10- and 16-month assessment points (Fiske et al., 2022, Fiske et al., 2024); the full administration protocols for both versions of the ECITT are available here. The blocked ECITT (with fNIRS) was completed in the second test session at all assessment points. Participants were seated on the lap of their caregiver and positioned at a table, adjacent to the experimenter. Whilst the cap was being fitted, participants watched a child-friendly cartoon on a laptop to keep them entertained and to ensure their head was straight and as still as possible during fitting. Before the task and fNIRS recording were started, the cartoon was paused, and the laptop was moved out of sight and reach of the child. There were no demonstration or practice trials. The first trial was always cued by the experimenter with the instruction “can you touch the happy face?” (later excluded from analysis). Verbal encouragement was given when necessary to increase engagement but was kept to a minimum. The experimenter removed the iPad from the participant’s reach (although still within their sight) during the animations that occurred after each trial and during baseline blocks to minimise the occurrence of premature or accidental responses.

### 2.5 Data processing

#### 2.5.1 Behavioural data processing

A sample of 21 videos was manually coded using the same coding scheme as in previous assessment points. Excellent inter-coder reliability (between a human coder and the data recorded by the app) was obtained for accuracy (Cohen’s kappa =.960) and reaction time (RT) correction (correct, valid trials only; Cohen’s kappa =.996). Note that it was only necessary to correct RT on eight trials (out of 1314 trials) and this was for eight different participants. This happened in instances where the iPad recorded the response time of the participant’s first touch, but the coder deemed that the first touch was accidental. As such, it was decided that the automated iPad-recorded accuracy and RT values were as reliable as manual coding of motor responses at 3½ years. The validity of the trials at 3½ years was also assessed by the coder during manual video coding. However, only one reason to exclude a trial was identified across all 21 videos, which was that the first trial was cued by the experimenter (as part of the protocol). Since this is consistent across participants, and the first trial is always excluded from analyses, it was decided that it was not necessary to manually code the validity of the trials in the ECITT at 3½ years. The accuracy and reaction time data reported in our analyses are therefore based on values (at the trial-level) recorded directly by the ECITT app (see section ‘Data and Code Availability’ for details about the app). As in previous work (Fiske et al., 2022, Fiske et al., 2024) we used mean prepotent accuracy, mean inhibitory accuracy, and the accuracy inhibitory score as dependent variables. The accuracy inhibitory score was used as an index of response inhibition performance such that better response inhibition was indicated by a higher accuracy inhibitory score. In accordance with the pre-registration, accuracy inhibitory score was calculated as: (1 – (mean prepotent accuracy – mean inhibitory accuracy)/mean prepotent accuracy). In the small number of cases where mean inhibitory accuracy > mean prepotent accuracy, accuracy inhibitory score was calculated as: (1 – (mean prepotent accuracy – mean inhibitory accuracy) × mean prepotent accuracy).

#### 2.5.2 fNIRS data processing

The Gowerlabs NTS system measured a raw intensity signal (780 and 850 nm) that underwent several transformations during pre-processing in HomER2 (Huppert et al., 2009), see Supplementary Materials 2.3. for a full description of the preprocessing pipeline. Trial blocks were excluded if the duration was longer than the mean duration + 2 SD for each block type (Control = 26.5 s, Experimental = 31.5 s). One block was excluded from 13 participants and two blocks were excluded from 2 participants for this reason. Following the pruning of poor-quality channels, participants with more than 2/3 of channels excluded were not included in further analysis (*N* = 13). Channels were excluded if less than 50 % of participants with valid fNIRS data contributed good-quality data (*N* = 10). More information about the location of these channels and possible reasons for poor data quality are provided in Supplementary Materials 7.2. Oxygenated haemoglobin (HbO_2_) and deoxygenated haemoglobin (HHb) concentration change data from each channel were block averaged over 22 seconds (containing two seconds of data from valid baseline blocks and 20 seconds of data from valid task blocks). Baseline concentration change data (two seconds) were subtracted from the average haemoglobin concentrations in the 20-second experimental window and the baseline-corrected data were divided into five four-second time bins. Following pre-processing and block averaging of the fNIRS data, average haemoglobin concentration data were available for each: individual (N = 61), chromophore (HbO_2_, HHb), channel (N = 36), condition (control, experimental, difference (control – experimental), and time bin (N = 5). See Supplementary Materials 2.6. for a detailed overview of the fNIRS preprocessing and analysis pipeline.

#### 2.5.3 Optode registration

Before submitting the pre-registration, the anatomical labels of brain regions covered by channels in the probe at 3½ years were generated by co-author Dr Liam Collins-Jones to support the predictions of effects in specific channels and brain regions. Please see Supplementary Materials 2.5 for the anatomical labels of all channels in the probe. As in our previous publications (Fiske et al., 2022, Fiske et al., 2024), the 12-month-old atlas from (Shi et al., 2011) was used for the head modelling. Whilst we recognise that there is a substantial change in head size from 12-months to 3½ years, we believe that the consistency gained from keeping the atlas the same throughout the three studies is advantageous and makes developmental comparisons more straightforward. This approach means that the channel localisation remained relatively consistent across studies, making it easier to investigate changes in activation over time in this longitudinal dataset. Further, we struggled to find an atlas that has suitable parcellation for 3½-year-olds, and we deemed that it would not be ideal to use an adult atlas with this young population. This is because a preliminary assessment based on our probe and an adult atlas (AAL2; Rolls et al., 2015) indicated that a large proportion of channels would be located in different cortical areas. Upon careful inspection of these adult-based locations and images of our probe placement, we concluded that they did not represent the channel locations accurately.

### 2.6 Study hypotheses and analysis approach

**Fig. 3:**
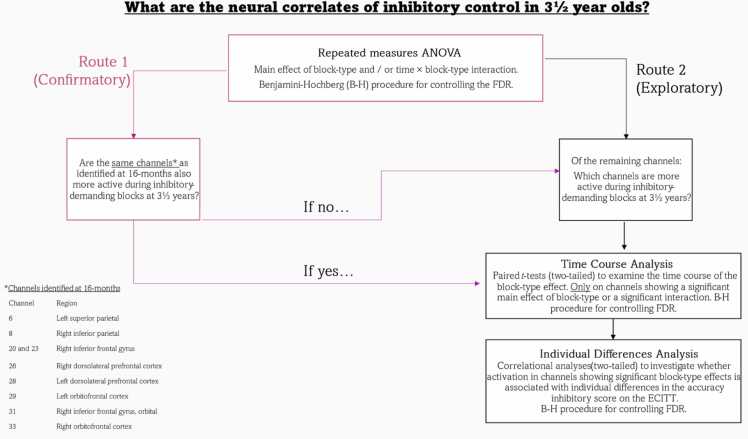
Pre-registered analysis plan for fNIRS analyses. Note. This flow chart diagram from our pre-registration illustrates the analysis plan for the fNIRS data. The two routes show the analysis approach we would take based on the outcome of the repeated measures ANOVA. Route 1 is confirmatory, based on the results of our previous work with 16-month-old infants (Fiske et al., 2024). Route 2 is exploratory and we will conduct the analyses on all remaining channels in the fNIRS probe.

The hypotheses, variables, and analysis plans were defined and pre-registered before analysis. All hypotheses relate to data from the 3½ year assessment point, unless explicitly stated as longitudinal. These are described in full in our pre-registration and are summarised in Supplementary Materials 3. Fig. 3 displays a flow chart overview of the pre-registered analysis plan for the fNIRS data.

Knowledge about the results from previous studies with this group of participants at 10- and 16-months (Fiske et al., 2022, Fiske et al., 2024) was used to form the pre-registered hypotheses about the current dataset. Similarly, since data from the behavioural ECITT (Session 1) at 3½ years was analysed for another study (pre-registered here), it was possible to use this to form hypotheses about the blocked ECITT performance at 3½ years. The behavioural ECITT (Session 1) data at 3½ years were analysed to examine between-session consistency and test re-test reliability where data from both versions of the ECITT are needed (results reported in Supplementary Materials 5.2.2. and 5.2.3). When behavioural data are mentioned in the current paper, this refers to the *blocked ECITT during the fNIRS session*. A brief description of our pre-registered hypotheses and planned analyses are provided in the paragraphs below.

*Behavioural Hypotheses:* Paired *t*-tests were conducted to test the hypotheses that 3½-year-old participants will be significantly more accurate, and respond significantly faster, on prepotent trials than on inhibitory trials.

*Longitudinal Behavioural Hypotheses:* Participants will be significantly more accurate on both trial types and have significantly better response inhibition performance at 3½ years than at 10-months and at 16-months. There will be a significant main effect of trial type (greater accuracy on prepotent compared to inhibitory trials), a significant main effect of age (greater accuracy at 3½ years than at 10-months), and a significant interaction (larger improvement in inhibitory accuracy than in prepotent accuracy between 10-months and 3½ years). The same prediction was made when examining accuracy development from 16-months to 3½ years. For RT from 16-months to 3½ years, we predicted a significant main effect of trial type (faster responses on correct prepotent compared to on correct inhibitory trials), no significant main effect of age, and no significant interaction. 2 × 2 linear mixed models will be conducted with trial type (prepotent, inhibitory) and age (10-months or 16-months and 3½ years) as within-subjects factors. We did not expect for accuracy inhibitory performance to be significantly correlated at 10-months and 3½ years, or at 16-months and 3½ years. Similarly, we did not expect a significant correlation between the median inhibitory RT at 16-months and 3½ years, although we did expect there to be a significant correlation between median prepotent RT at 16-months and 3½ years. Correlational analyses were conducted to test these hypotheses.

*fNIRS Hypotheses:* It was predicted that the haemodynamic response in nine channels overlying regions of the PFC and parietal cortex would be differentiated by block type (i.e., *experimental blocks* requiring inhibition vs. *control blocks* with no inhibitory requirement). These channels were: Channel 6 (left superior parietal), Channel 8 (right inferior parietal), Channels 20 and 23 (right IFG), Channel 26 (right DLPFC), Channel 28 (left DLPFC), Channel 29 (left OFC), Channel 31 (right IFG, orbital), and Channel 33 (right OFC). Once this hypothesis was tested, we conducted exploratory analyses on the remaining channels in the probe to investigate whether any other channels were showing significant block type effects. Data were divided into five 4-second time bins (0 – 20 seconds of the block time course). Repeated measures ANOVAs were conducted for each channel and each chromophore (HbO_2_ and HHb) with time bin (5 levels) and block type (2 levels) as within-subjects factors. Channels showing a significant main effect of block type, or a significant time × block type interaction in either chromophore were considered as showing a significant block type effect. If channels showed significant block type effects, two-tailed paired t-tests were conducted to investigate the time course of the significant effect (these results are reported in Supplementary Materials 7.2.).

*Individual Differences (Brain-Behaviour) Hypotheses:* Since findings from the 10-month dataset relating to potential associations between individual performance differences and inhibition-specific brain activation were weak and did not survive correction for multiple comparisons (Fiske et al., 2022), and there were no significant associations in the 16-month dataset (Fiske et al., 2024), no specific predictions were made about whether there would be significant brain-behaviour associations at 3½ years. Two-tailed correlational analyses were conducted to investigate whether individual differences in neural activation in these channels (across the time bins showing significant effects) were associated with individual differences in the accuracy inhibitory score.

*Neural Development from Infancy to Early Childhood:* We made no specific predictions about the neural development from infancy (10-months, 16-months) to early childhood (3½ years). As such, exploratory linear mixed model analyses were conducted to investigate the change in activation in each channel showing significant block type effects across assessment points. Two separate analyses (Model 1: 10-months to 3½ years, Model 2: 16-months to 3½ years) were conducted on channels showing significant block type effects at the 10-month and/or 16-month assessment points, and any new channels identified at 3½ years. Results of these pre-registered exploratory analyses are reported in Supplementary Materials 7.6.

### 2.7 Statistical analysis

All statistical analyses were conducted in SPSS version 29. All variables were tested against the parametric test assumptions, and when violated, appropriate non-parametric equivalents were used. Greenhouse-Geisser corrected degrees of freedom and significance values were used because the channel-level fNIRS data did not meet the sphericity assumption required for repeated measures ANOVA. The procedure for controlling the false discovery rate (FDR) (Benjamini and Hochberg, 1995) was used where multiple tests were conducted. CIs (95 %) were calculated on 1000 bootstrap samples. Please see Supplementary Materials 4 for details of how the data were tested against the parametric test assumptions and Supplementary Materials 6 for the results of any equivalent non-parametric tests that were conducted.

## 3 Results

### 3.1 Behavioural results

**Trial type effects:** Results of paired samples *t*-tests revealed that 3½-old participants (*N* = 100) were significantly more accurate on prepotent trials compared to inhibitory trials: *t* (99) = -9.404, *p* < .001, *d* = -.940, and were significantly quicker at responding on correct prepotent trials than on correct inhibitory trials: *t* (99) = 15.729, *p* < .001, *d* = 1.573.

**Fig. 4:**
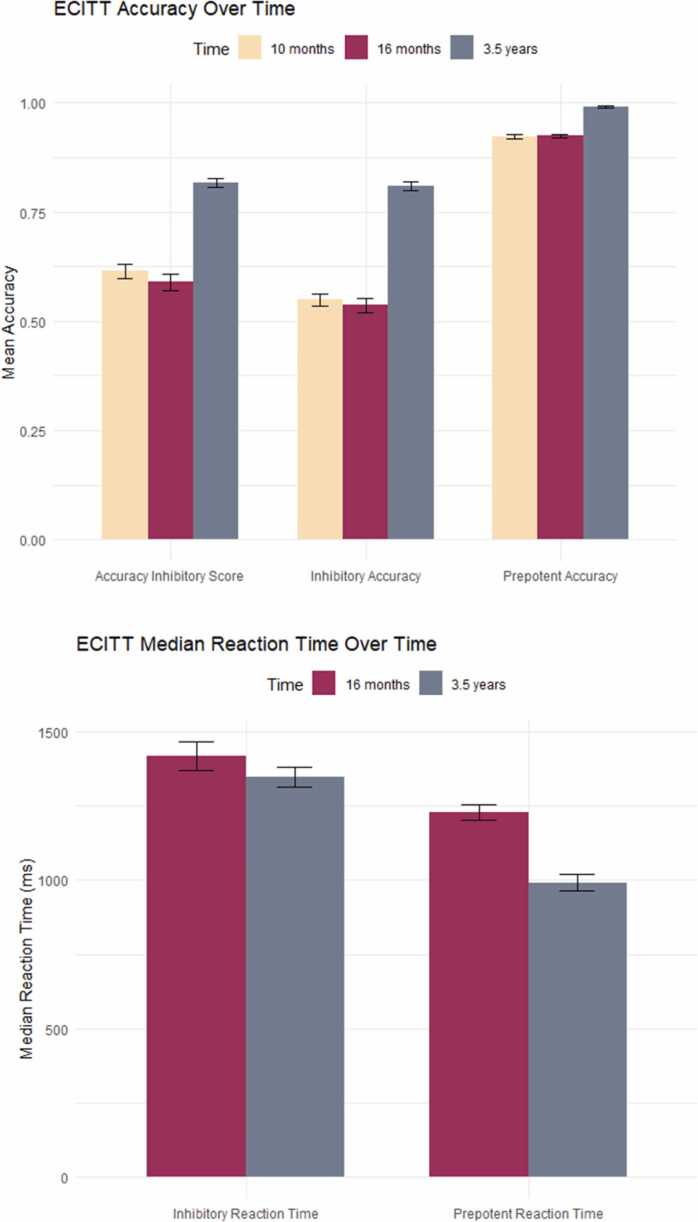
Behavioural development on the ECITT from infancy to early childhood. Note. Error bars represent standard error. Top figure displays the mean accuracy on the two trial types and the Accuracy Inhibitory Score on the ECITT at 10-months, 16-months and 3½ years. Bottom figure displays the median reaction time (ms) data for the two trial types on the ECITT at 16-months and 3½ years.

**Behavioural Change from Infancy to Early Childhood:** Descriptive statistics for the ECITT performance variables at 10-months, 16-months and 3½ years are reported in Table 2 below. See Fig. 4 for a visualisation of behavioural (accuracy and RT) change on the ECITT from infancy to early childhood.

**Table 2:** Descriptive statistics for longitudinal ECITT performance.

	10-months		16-months		3½ years	
	N	Mean (SD)	N	Mean (SD)	N	Mean (SD)
Mean inhibitory accuracy	128	.549 (.292)	81	.536 (.324)	100	.810 (.199)
Mean prepotent accuracy	128	.922 (.085)	81	.924 (.088)	100	.991 (.039)
Accuracy inhibitory score	128	.615 (.337)	81	.590 (.369)	100	.817 (.194)
Median inhibitory RT (ms)	-	-	77	1454 (418)	100	1389 (328)
Median prepotent RT (ms)	-	-	77	1280 (217)	100	1024 (280)

Note. Larger accuracy inhibitory score = better response inhibition ability.

Results of two paired samples *t*-tests revealed that there was a significant improvement in performance on the ECITT (accuracy inhibitory score) between 10-months and 3½ years: *t* (77) = -4.246, *p* < .001. *d* = -.481, *N* = 78, and between 16-months and 3½ years: *t* (43) = -2.817, *p* = .004, *d* = -.425, *N* = 44. No significant longitudinal associations were found between performance on the ECITT in infancy and early childhood (results are reported in Supplementary Materials 5.2.1).

Results of linear mixed models (restricted maximum likelihood estimation) (Table 3) revealed a significant main effect of age, such that participants were significantly more accurate, and responded significantly faster, at 3½ years than at 10-months of age or 16-months of age. A significant main effect of trial type was found in both models whereby participants were significantly more accurate, and responded significantly faster, on prepotent trials than on inhibitory trials. A significant interaction effect was observed in both models, such that there was a larger improvement in inhibitory accuracy than prepotent accuracy between 10 months and 3½ years (Model 1) and between 16-months and 3½ years (Model 2). There was also a significant interaction effect for the RT data, such that there was a larger RT improvement in prepotent RT between 16-months and 3½ years than in inhibitory RT. See Supplementary Materials 5.1 for estimated marginal means.

**Table 3:** Results of linear mixed models (Accuracy and RT) on the ECITT from infancy to early Childhood.

Model 1: (10-months and 3½ years)	Test Statistic	*p*	Effect size (Cohen’s *f*)
Trial type	*F* (1, 247.289) = 265.532	< .001	1.03
Age	*F* (1, 247.289) = 94.454	< .001	0.366
Interaction	*F* (1, 247.289) = 32.007	< .001	0.353
Model 2: (16-months and 3½ years)	Test Statistic	*p*	Effect size (Cohen’s *f*)
Trial type	*F* (1, 143.137) = 179.597	< .001	1.11
Age	*F* (1, 143.137) = 64.598	< .001	0.662
Interaction	*F* (1, 143.137) = 23.837	< .001	0.397
Model 3: RT (16-months and 3½ years)	Test Statistic	*p*	Effect size (Cohen’s *f*)
Trial type	*F* (1, 248.311) = 21.615	< .001	0.287
Age	*F* (1, 248.311) = 61.126	< .001	0.490
Interaction	*F* (1, 248.311) = 7.751	.006	0.164

*Note.* Model 1: *N* = 146, Model 2: *N* = 137, Model 3: *N* = 135

### 3.2 fNIRS group-level results

**Fig. 5:**
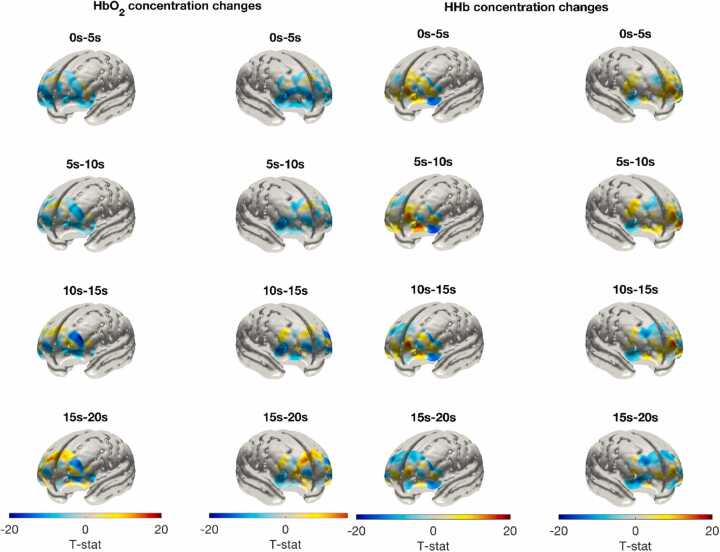
Group-level T-Statistic Image of the Contrast in Haemoglobin Concentration Changes Between Block Types at 3½ years. Note. Group-level T-statistic images of the contrast in concentration changes of each chromophore (HbO_2_ on the left and HHb on the right) between experimental and control blocks (experimental – control) in the left hemisphere (left image) and right hemisphere (right image). This figure was produced by comparing concentration changes (in both HbO_2_ and HHb) at each node in the reconstructed image in equivalent 5 s time-bins between the experimental and control conditions with a two-sample *t*-test. A threshold of p < 0.01 was used to indicate significance. Images are displayed in the space of a cortical surface derived from averaged structural MRI data of a 12-month-old cohort of infants (Shi et al., 2011). Data were contributed by 61 participants, although not all participants contributed valid data to each channel. Images produced by co-author LCJ.

**Fig. 6:**
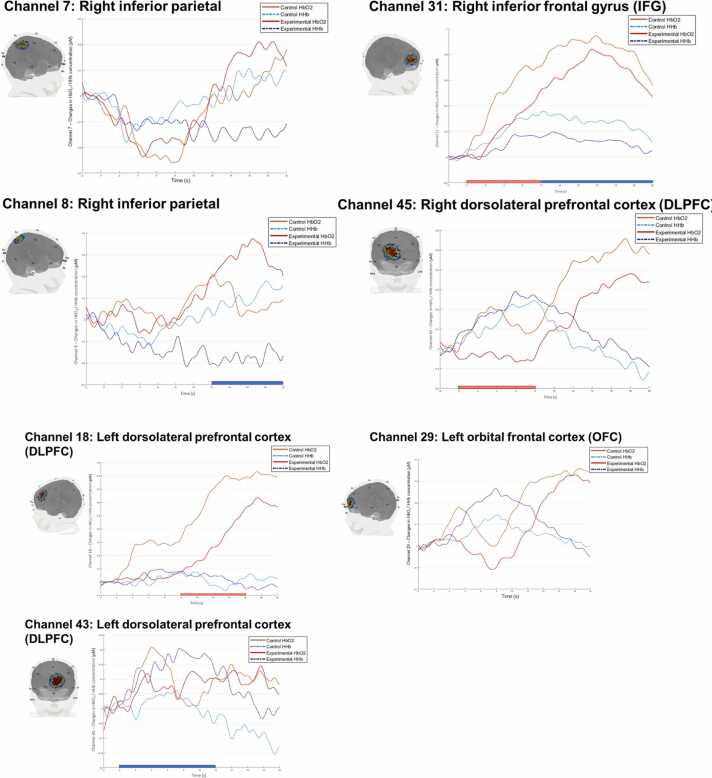
Haemodynamic Response Function for Channels Showing Significant Block Type Effects at 3½ years. *Note.* This figure illustrates the average haemodynamic response function from −2 s (baseline) to 20 seconds of the block time course for channels showing significant block type effects. The change in concentration in HbO_2_ and HHb (μM) for each channel are plotted on the y-axis, time bin is plotted on the x-axis. Bars (blue = HHb, red = HbO_2_) on the x-axis indicate the time-bins where there is a significant block-type effect (note that these time-bin effects did not survive the correction for the FDR but are added here for illustrative purposes). Sensitivity profiles illustrate the position of each channel on the cortex and the channel sensitivity (heat map: red = more sensitive, blue = less sensitive). Data were contributed by 61 participants, although not all participants contributed valid data to each channel.

Valid fNIRS data were contributed by 61 participants at the 3½ year assessment point. A *t*-statistic image of significant haemoglobin concentration differences between block types is shown in Fig. 5 (for visualisation purposes). See also Fig. 6 for HRF plots for all channels showing a significant block type effect. A detailed description of the significant block type effects can be found in Supplementary Materials 7.3.

**Confirmatory analyses:** Repeated measures ANOVAs were conducted on data from eight channels (note that nine channels were pre-registered, but a channel covering the left superior parietal cortex (Channel 6) was excluded as fewer than 50 % of participants contributed data to this channel). Seven channels demonstrated a significant main effect of time (haemoglobin concentration change from baseline; results are reported in Supplementary Materials 7). All channels retained significance for the main effect of time when controlling for the FDR. Three channels covering the right inferior parietal cortex (Channel 8), the left OFC (Channel 29) and right IFG (Channel 31) showed significant block type or time × block type interaction effects (Table 4). This suggests that activation in these channels significantly differed by block-type.

**Table 4:** Significant HbO_2_ and HHb concentration change differences between block types at 3½ years.

Location	Hemisphere	Channel	Signal	Statistic
Inferior parietal	Right	7	HHb	*F* (1.656, 57.964) = 4.219, *p* = .026, ηp^2^ = .108, Cohen’s *f* = .297
		8 *	HHb	*F* (1.600, 51.189) = 3.954, *p* = .034, ηp^2^ = .109, Cohen’s *f* = .296
IFG	Right	31 *	HbO_2_	*F* (1, 51) = 4.554, *p* = .038, ηp^2^ = .082, Cohen’s *f* = .259
			HHb	*F* (1, 51) = 5.423, *p* = .024, ηp^2^ = .096, Cohen’s *f* = .289
DLPFC	Left	18	HbO_2_	*F* (1, 49) = 6.400, *p* = .015, ηp^2^ = .116, Cohen’s *f* = .325
		43	HHb	*F* (1, 40) = 5.859, *p* = .020, ηp^2^ = .128, Cohen’s *f* = .340
	Right	45	HbO_2_	*F* (1, 46) = 5.999, *p* = .018, ηp^2^ = .115, Cohen’s *f* = .323
OFC	Left	29 *	HHb	*F* (2.159, 120.920) = 3.142, *p* = .043, ηp^2^ = .053, Cohen’s *f* = .193

*Note.* * = Channels were pre-registered in the confirmatory analyses (and showed significant effects at 16-months). The effect found in Channel 29 was a significant time x block-type interaction, whereas all others were main effects of block type. None of the effects survived the procedure for controlling the FDR (Benjamini and Hochberg, 1995). IFG = inferior frontal gyrus, DLPFC = dorsolateral prefrontal cortex, OFC = orbital frontal cortex. Data were contributed by 61 participants, although not all participants contributed valid data to each channel.

**Exploratory analyses:** Repeated measures analyses were conducted on the remaining channels in the fNIRS probe to examine whether any additional channels (to those pre-registered) showed significant block type effects or interactions. Note that data from 9 additional channels were excluded from the analyses as fewer than 50 % of participants contributed data (for more information see Supplementary Materials 7.2). Of the 25 channels included in the exploratory repeated measures analyses, 24 channels demonstrated a significant main effect of time in the HbO_2_ and/or the HHb signal (results are reported in Supplementary Materials 7.1). Following correction for the FDR, there were only three channels for which the main effect of time was no longer significant. Four channels covering the right inferior parietal cortex (Channel 7), the left DLPFC (Channels 18 and 43) and the right DLPFC (Channel 45) were found to show a significant block type effect or a significant time × block type interaction effect (Table 4). This suggests that activation in these channels significantly differed by block-type.

**Time course analysis:** Paired *t*-tests were conducted on the seven channels (listed in Table 4) identified as showing significant block-type effects. Full results are reported in Supplementary Materials 7.3. Fig. 6 illustrates the time bins showing significant block type effects which are highlighted in each channel with either a red (HbO_2_) or blue (HHb) bar. Note that for channels covering the right inferior parietal cortex (Channel 7) and the left OFC (Channel 29), there were no significant HHb concentration differences between block types within any of the five time bins. This is because exploratory paired *t*-tests were conducted using the two-tailed *p*-value.

### 3.3 Individual differences (brain-behaviour associations)

Results of Pearson’s correlational analyses (reported in Supplementary Materials 7.4) revealed that there were no significant associations between performance on the ECITT and the haemoglobin difference variables in any of the channels found to be showing significant block-type effects.

### 3.4 Neural development from infancy to early childhood

Since no channels showed significant block type effects at both 10-months and 3½ years, it was decided that the analyses examining change from 10-months to 3½ years would not be conducted (deviating from the pre-registered analysis plan). However, we did conduct pre-registered exploratory longitudinal analyses to examine how brain activation changes from 16-months to 3½ years in channels showing significant block type effects. Results are reported in Supplementary Materials 7.6 and are preliminary as they did not survive the FDR correction.

## 4 Discussion

### 4.1 Response inhibition development from infancy to early childhood

In line with previous research in infants and toddlers (Fiske et al., 2022, Fiske et al., 2024, Hendry et al., 2021), the current study showed that 3½-year-olds are significantly more accurate and respond significantly faster on prepotent trials than on inhibitory trials. This demonstrates that the preschool version of the ECITT, which is more difficult due to faster trial presentation, provides an inhibitory challenge for 3½-year-olds, and as such, is an appropriate response inhibition task for this age group.

There was a significant improvement at the group-level in accuracy and RT on both trial types at 3½ years compared to the younger ages. There was a larger accuracy improvement on inhibitory trials, suggesting that inhibitory control skills develop significantly across the transition to early childhood (as also found by Holmboe et al., 2021). However, the larger RT improvement was on prepotent trials, suggesting that 3½-year-olds are now quick and accurate at responding on prepotent trials but still need to slow down substantially on inhibitory trials to produce the correct response. This could be reflective of a speed-accuracy trade-off at 3½ years, where children are better able to slow down their responses on inhibitory trials to avoid errors, whereas 16-month-olds are still responding more impulsively (Fiske et al., 2024).

No significant associations were found when examining longitudinal stability in individual performance differences from infancy to early childhood. Since evidence supports stability in individual differences from around 24-months (e.g., Broomell and Bell, 2022), it was not expected that performance would be significantly correlated from infancy to early childhood.

### 4.2 The neural correlates of response inhibition from infancy to early childhood

The current study, along with our previous work, highlights the change in prefrontal and parietal cortex activation when inhibition is required on the same task from infancy to early childhood. At 10-months, activation is localised to right-lateralised regions of the superior parietal cortex and dorsal regions of the PFC (Fiske et al., 2022). By 16-months, activation is now found bilaterally across the DLPFC and OFC and we also see the involvement of the left superior parietal cortex, as well as the right inferior parietal cortex (Fiske et al., 2024). Additionally, we previously found that 16-month-olds activate the right IFG when inhibition is required – an area of the brain that has been pinpointed as a key neural substrate for inhibition (Aron et al., 2014, Chikazoe et al., 2007, Hampshire et al., 2010). In the current study at 3½ years, children recruited the right inferior parietal cortex and the right IFG in inhibitory demanding blocks. The consistent recruitment of these right-lateralised brain regions across infancy and early childhood may be indicative of the maturation of the neural system that underpins early response inhibition.

#### 4.2.1 Unexpected findings at 3½ years

There were also some unexpected findings related to neural activation in some channels overlying the left OFC and the bilateral DLPFC. Of note, these effects were in the *opposite direction* from what would be expected if the areas were involved in response inhibition, i.e., activation was greater in control blocks (where there is no inhibitory demand) than in experimental blocks (where there is an inhibitory demand). We did not see this pattern of activation at 10- or 16-months (Fiske et al., 2022, Fiske et al., 2024). Therefore, we do not believe that this activation reflects response inhibition processes, however, we still need to consider why this activation occurs and why we observe it only when children reach preschool age.

The role of the left OFC appears to change from infancy to early childhood during the ECITT, as whilst it is not recruited at 10-months, this region was recruited during experimental (inhibitory-demanding) blocks at 16-months, and by 3½ years, is more active during control blocks with no inhibitory demand. However, the results of the current study suggest that the role of the OFC is unrelated to perseveration or inhibitory processes at 3½ years, since the control blocks do not contain any response inhibition demands. During the ECITT we have also seen the consistent recruitment of the DLPFC from infancy to early childhood. However, at 3½ years, this unexpected activation was more dorsal and closer to the midline, and in the opposite direction, than the DLPFC activation we have observed in our previous work at younger ages in the same sample (Fiske et al., 2022, Fiske et al., 2024).

Unlike infants and toddlers, perhaps 3½-year-olds are exerting proactive control during control blocks. According to (Braver, 2012), top-down proactive control supports the maintenance of task goals in preparation for upcoming task requirements which have a higher cognitive demand (such as the need to inhibit in experimental blocks of the ECITT). Indeed, the DLPFC has been evidenced to be a key neural substrate in proactive control (Aron, 2011, Hwang et al., 2014, Hwang et al., 2016). Also, it may be that there is greater involvement of the DLPFC for 3½ year-olds because they are engaging in other cognitive processes (e.g., working memory, monitoring) to support them in maintaining task rules in control blocks in preparation for the more challenging experimental blocks (Chatham et al., 2012). A review by Chevalier (2015) proposes that the development of EF in children comes with the increased recruitment of proactive control processes to effectively meet task demands (see also, Chevalier et al., 2015). As such, it is possible that 3½ year-olds are already engaging in rudimentary proactive control processes that represent a pivotal milestone in the development of mature inhibitory control (in contrast to more moment-to-moment response inhibition processes in infancy and toddlerhood).

Another potential explanation for the unexpected activation observed at 3½ years could be to do with the trivially easy nature of the control blocks at this age (i.e., repeatedly touching the target in the same place on the screen). It could be that 3½-year-olds have to engage in more active attentional control (i.e., concentration, ignoring distractions) during control blocks than experimental blocks to keep going with the task. They are also more likely to have an awareness that continuing with the task (even when bored) is what is expected of them. In contrast, the experimental blocks required children to inhibit a prepotent response on some trials, which may engage their attention without much extra effort due to the increased cognitive demand. (Halliday et al., 2018) highlighted six factors that underpin task engagement in preschool children (attention to instructions, on-task behaviour, persistence, monitoring progress / strategy use), many of which could be argued to require broader executive processes (that may recruit the DLPC), in contrast to response inhibition per se.

To test these post-hoc theories, the task procedure could be tweaked to prevent children from engaging in meta-cognitive or other executive processes specifically in the very easy (for this age group) control blocks. Future research using the ECITT with 3½-year-olds could impose a time demand on trials by asking participants to respond as quickly and accurately as possible, as we have done in previous behavioural studies with older children and adults (Holmboe et al., 2021). This would increase the task demands in control blocks, leaving less space for other off-task cognitive processes. Further, the presentation order of blocks could be randomised such that they cannot be predicted. If task blocks are randomised, and quick and accurate responses are encouraged, we may predict that the greater activation seen in the dorsal/midline PFC and the OFC during control blocks in the current study disappears. This is because the child would not be able to predict whether the next block is a control or experimental block, so the need for proactive control would be the same across all blocks.

### 4.3 Limitations and future directions

It cannot be ignored that there is a large gap in time between our 16-month time point and our 3½-year time point. Although this was unplanned and caused by COVID-19 pandemic-related lab closures, it is worth noting that a significant amount of development occurs between those two ages. Unfortunately, this development is not captured by our current dataset. We are keen to collect new longitudinal data that measures development across this critical toddlerhood period. We already know that 24-months of age is a pivotal point for stability in early EF skills (Broomell and Bell, 2022, Holmboe et al., 2021), and so we expect that data collected during this time would help us fill in the missing piece of the puzzle when building a neurocognitive developmental trajectory of early inhibitory control development. Additionally, we are aware that we could potentially uncover more answers about the neural correlates of early inhibitory control by examining functional connections between the prefrontal and parietal cortices. Whilst this was beyond the scope of the current study (which aimed to examine activation in specific regions of interest), future research is planned that adapts functional connectivity measures to the task design used in the current study, such that the involvement of functional networks can potentially be uncovered.

Many of the significant effects reported in the fNIRS analyses did not survive the procedure for controlling the FDR (Benjamini and Hochberg, 1995), and therefore requires replication. There was also a lack of statistical power for the longitudinal analyses due to the large amount of attrition at 16-months (due to necessary lab closures during the COVID-19 pandemic) and the sample size at 3½ years being smaller than intended. As such, the number of participants who contributed valid behavioural and/or fNIRS data to at least two assessment points is fairly low. Nonetheless, rigorous protocols were put in place to ensure that transparent scientific procedure was followed (such as pre-registering the hypotheses and analysis plans in great detail and taking a stringent multiple comparisons correction approach to minimise the risk of Type I errors). This will support future efforts to build on this work.

Finally, whilst significant changes in head size and structural brain development occur from infancy to early childhood, we decided to use the same head model (12-month AAL atlas; Shi et al., 2011), such that more reliable developmental comparisons could be made as the channel localisation remains relatively consistent across assessment waves. However, some of the subtle changes in which channels are activated (such as activation moving to a neighbouring channel) could be due to the changes in head size at 3½ years. Future research is needed both in the development of freely available MRI atlases with appropriate parcellation for developmental populations, and also to account for the change in head size and brain structure and anatomy in early developmental longitudinal research (although note that a possible solution has recently been proposed by Magee et al. (2023).

### 4.4 Strengths and implications

Despite the sample attrition discussed in the previous section, the longitudinal data presented in this and our previous studies from the three assessment points across infancy and early childhood constitute a great asset. The sample size at individual ages (N = 43–61) was large for an early childhood fNIRS study, and we believe that our findings will be very valuable to the field. Not only has this longitudinal study enabled the investigation of developmental change across a currently under-studied period of early life, but it has also contributed new knowledge about the neural correlates of response inhibition from its emergence in infancy and as it matures into early childhood. As such, we now know the areas of the prefrontal and parietal cortices that are involved in inhibitory control across the period from infancy to pre-school, including the substantial changes in the recruitment of these brain regions across this period.

An additional strength of this study is that the experimental paradigm and methodological approach remained the same across all assessment points. Due to difficulties in finding inhibitory control tasks that are suitable for use with infants and young children, it is very rare for longitudinal studies to continue to use the same task across assessment points spanning infancy and early childhood. Our work has demonstrated that the ECITT is an appropriate and reliable measure of response inhibition from as young as 10-months of age (Fiske et al., 2022; see also, Hendry et al., 2021), can continue to be used in early toddlerhood (Fiske et al., 2024) and, with minor modification, is also a reliable measure of response inhibition at 3½ years of age. By using the same task across assessment points, we can be confident that we are observing age-related performance improvements, rather than a methodological artifact related to differing task demands. Additionally, given the simplicity of the ECITT, it is our hope that our methods can be used in future research to look at inhibitory control development in groups of children at risk of developmental disorders such as Autism and ADHD.

Finally, by also keeping the fNIRS methodology identical between assessment points (i.e., same cap, same processing pipeline, same head model for optode localisation), more reliable comparisons of change in neural activation could be made across assessment points, something that has rarely been done (particularly with the same cohort of participants and the same manual response task) in the developmental fNIRS literature. Overall, the findings from the current study contribute exciting new knowledge about the neural correlates of response inhibition across the early years of life and begin to piece out the trajectory from the emergence of rudimentary inhibitory skills in infancy to more mature skills in early childhood.

## 5 Conclusion

The current study presents novel longitudinal data on age-related changes in the neural correlates of response inhibition from infancy to early childhood. Our previous work found that whilst response inhibition in infancy (at 10 months) appears to be supported by right-lateralised regions of the prefrontal and parietal cortices (Fiske et al., 2022), the transition to toddlerhood (at 16 months) sees the recruitment of bilateral prefrontal regions and of the left parietal cortex to support response inhibition, despite there being no behavioural improvements in response inhibition (Fiske et al., 2024). In the current study (at the start of early childhood; 3½ years), a clear improvement in response inhibition performance is observed and some of the same cortical regions as identified at 16-months are still being recruited when inhibition is needed, specifically the right inferior parietal cortex and the right IFG. This consistent recruitment suggests that these brain regions are fundamental neural indices of inhibitory control, even from infancy when inhibitory skills are still emerging. Children also recruited additional areas during task performance, specifically areas of the DLPFC that were more dorsal and closer to the midline than at younger ages. Because the activation in these areas was stronger when response inhibition was *not* required, we propose that this activation may reflect other cognitive processes, which will need further investigation in future research.

## Funding

This work was supported by the UK Medical Research Council (MR/N008626/2, PI: KH) and by AF's UK Medical Research Council Industrial Collaborative Awards in Science and Engineering (iCASE) studentship. The funder was not involved in the conceptualisation, design, data collection, analysis, decision to publish, or preparation of the manuscript.

## Data and code availability

The data that support the findings of this study and the custom MATLAB scripts used to analyse the fNIRS data are available on the Open Science Framework: https://osf.io/n7zyx/. The code for the original ECITT task and the blocked version of the ECITT used with fNIRS are available on Figshare. Please contact Dr Karla Holmboe for details on how to access demo versions of both tasks.

## Author contributions

KH conceptualised the idea for the study and the original ECITT task design, and KH, CdK and HD designed the fNIRS version of the ECITT. AF, CdK, LCJ and KH formulated the analysis plan and wrote the pre-registration. AF, AM and SUG collected the 3½-year data (contributions to the infant and toddler data collection are provided in Fiske et al. 2022 and Fiske et al. 2024). AF, AM, KH and HD curated the data. AF, AM and CdK processed and analysed the fNIRS data. AF, CdK and KH conducted the statistical analyses. LCJ and AF visualised the data. HD programmed the ECITT software, and LCJ developed the pipeline to reconstruct the data and to display the resulting reconstructed images. AF wrote the original draft. AF, AM, KH, GS, CdK, and SUG reviewed and edited the original draft. KH and GS supervised the study, and KH undertook the overall management, project administration and acquired the funding for the larger project that this study was part of.

## CRediT authorship contribution statement

**de Klerk Carina C J M:** Writing – review & editing, Methodology, Formal analysis. **Collins-Jones Liam:** Visualization, Formal analysis. **Mortimer Alicia:** Writing – review & editing, Investigation, Data curation. **Fiske Abigail:** Writing – review & editing, Writing – original draft, Visualization, Project administration, Methodology, Investigation, Funding acquisition, Formal analysis, Data curation. **Holmboe Karla:** Writing – review & editing, Validation, Supervision, Resources, Project administration, Methodology, Investigation, Funding acquisition, Formal analysis, Data curation, Conceptualization. **Scerif Gaia:** Writing – review & editing, Supervision. **Dvergsdal Henrik:** Software, Resources, Data curation. **Gattas Sylvia Ulieta:** Writing – review & editing, Investigation.

## Declaration of Competing Interest

The authors declare that they have no known competing financial interests or personal relationships that could have appeared to influence the work reported in this paper.
