# Supplementary material for: Inhibitory control development from infancy to early childhood: A longitudinal fNIRS study
Source: Dev Cogn Neurosci. 2025 Mar 26;73:101557. doi: 10.1016/j.dcn.2025.101557 (PMC11997363; doi:10.1016/j.dcn.2025.101557)
Supplement: Supplementary file 1 — Supplementary material [file mmc1.docx]

**Inhibitory Control Development from Infancy to Early Childhood: A Longitudinal fNIRS Study**

**Supplementary Materials**

Abigail Fiske*, Alicia Mortimer, Liam Collins-Jones, Carina de Klerk, Sylvia Gattas, Henrik Dvergsdal, Gaia Scerif, Karla Holmboe

*Corresponding author: Dr Karla Holmboe, School of Psychological Science, University of Bristol, Bristol, United Kingdom. Email: [karla.holmboe@bristol.ac.uk](mailto:karla.holmboe@bristol.ac.uk)

Note. The following supplementary materials correspond to the article (as titled above). See also our project on the Open Science Framework [<https://osf.io/n7zyx>] for materials relating to the article, these supplementary materials, or the data to which these reports are associated. The materials in this project are under a CC-By Attribution 4.0 International license. Please cite the article if using any of these materials.

**Table of Contents**

[**1.** **Data Exclusions and Participant Demographics** 3](#_Toc173243796)

[**1.1.** **Data Exclusions** 3](#_Toc173243797)

[**1.2.** **Participant Demographics** 4](#_Toc173243798)

[**2.** **fNIRS: Array and Cap Placement, Pre-processing, Channel Locations, Analyses, Head Modelling and Image Reconstruction** 5](#_Toc173243799)

[**2.1.** **Visualisations of the fNIRS Array** 5](#_Toc173243800)

[**2.2.** **fNIRS Cap Placement** 7](#_Toc173243801)

[**2.3.** **fNIRS Pre-processing in HomER2** 7](#_Toc173243802)

[**2.4.** **Head Modelling, Channel Localisation and Image Reconstruction** 8](#_Toc173243803)

[**2.5.** **Anatomical Labels of Channels** 9](#_Toc173243804)

[**2.6.** **Overview of the fNIRS Preprocessing and Analysis Pipeline** 11](#_Toc173243805)

[**3.** **Pre-registered hypotheses, analysis plans and variables** 15](#_Toc173243806)

[**3.1.** **Pre-registered hypotheses and analysis plans** 15](#_Toc173243807)

[**3.2.** **Description of Variables** 21](#_Toc173243808)

[**4.** **Parametric Test Assumptions** 23](#_Toc173243809)

[**5.** **Additional Results** 25](#_Toc173243810)

[**5.1.** **Estimated Marginal Means for Linear Mixed Models** 25](#_Toc173243811)

[**5.2.** **Longitudinal Associations, Between-Session Consistency, Test Retest Reliability and Group Differences** 26](#_Toc173243812)

[**6.** **Results of Non-Parametric Tests** 30](#_Toc173243813)

[**7.** **fNIRS Group-Level Results** 35](#_Toc173243814)

[**7.1.** **Main Effect of Time** 35](#_Toc173243815)

[**7.2.** **Excluded Channels** 36](#_Toc173243816)

[**7.3.** **Time Course of the Significant Block-Type Effect** 37](#_Toc173243817)

[**7.4.** **Description of the Significant Block Type Effects** 38](#_Toc173243818)

[**7.5.** **Individual Differences (Brain-Behaviour) Analyses** 39](#_Toc173243819)

[**7.6.** **Longitudinal fNIRS Analyses** 40](#_Toc173243820)

[**8.** **References** 46](#_Toc173243821)

## **Data Exclusions and Participant Demographics**

### **Data Exclusions**

As per the Oxford Early Executive Functions (OEEF) study inclusion criteria, one female participant was excluded from the study due to their low birth weight, and three male participants were excluded due to birth complications leading to health-related concerns. Following this, 125 children contributed behavioural ECITT data (Session 1), 121 children contributed blocked ECITT (Session 2) data, and 100 children contributed fNIRS data to this study. The final sample sizes after data exclusions are reported in the manuscript and see **Supplementary Table 1** for details about exclusion criteria.

**Supplementary Table 1.** Data Exclusions.

| **Session 1 (behavioural ECITT)** | ***N*** |
| --- | --- |
| Experimenter administration error | 3 |
| Did not complete the full task | 2 |
| Software failure: data did not save | 1 |
| **Total Excluded** | **6** |
| **Percentage Excluded** | **4.8%** |
| **Total Included** | **119** |
| **Session 2 (blocked ECITT)** | ***N*** |
| Less than 2 blocks of each condition completed | 2 |
| Completed an earlier version of the task | 19 |
| **Total Excluded** | 21 |
| **Percentage Excluded** | **17.36%** |
| **Total Included** | **100** |
| **fNIRS Data** | ***N*** |
| Data file was lost | 1 |
| Refused to wear fNIRS cap | 10 |
| The fNIRS cap was removed before the minimum number of blocks had been completed | 2 |
| fNIRS cap placement was invalid | 5 |
| No longer had the minimum number of blocks following exclusion of invalid or extra-long blocks | 8 |
| Poor signal quality in > 30 channels | 13 |
| **Total Excluded** | 39 |
| **Percentage Excluded** | **39.0%** |
| **Total Included** | **61** |

### **Participant Demographics**

A total of 101 participants (N = 49 male) contributed at least some data (online questionnaire or in-person sessions) to the 3½ year assessment point of the OEEF preschool study. Of these, 94 participants (N = 42 male) attended the first in-person test session at the 3½ year assessment point, and 91 infants (N = 41 male) returned for their second test session approximately one week later. As per the OEEF study inclusion criteria (described above), two male infants were excluded from the study due to birth complications leading to health-related concerns. Demographic information for this sample is reported in **Supplementary Table 2** below. Demographic information for participants at the 10- and 16-month assessment points can be found in Fiske et al. (2022) and Fiske et al. (2024).

**Supplementary Table 2**. Demographic information for the 3½ year participant sample.

|  | *N* | Mean | SD | Min | Max |
| --- | --- | --- | --- | --- | --- |
| Child’s age at Session 1 (months) | 94 | 41.5 | 0.56 | 40 | 43 |
| Child’s age at Session 2 (months) | 91 | 41.87 | 0.56 | 41 | 44 |
| Mother’s age (years) | 93 | 36.93 | 4.28 | 27 | 50 |
| Father’s age (years) | 85 | 38.16 | 5.07 | 25 | 56 |
| Maternal education (years) | 92 | 18.35 | 2.86 | 12 | 30 |
| Paternal education (years) | 84 | 17.80 | 2.91 | 11 | 25 |
| **Infant Ethnicity** | *N* | % |  |  |  |
| Asian | 1 | 1 |  |  |  |
| Mixed – Other | 1 | 1 |  |  |  |
| Other White | 16 | 16 |  |  |  |
| Prefer not to say | 1 | 1 |  |  |  |
| White and Arabic | 1 | 1 |  |  |  |
| White and Asian | 3 | 3 |  |  |  |
| White and Black African | 1 | 1 |  |  |  |
| White and Black Caribbean | 1 | 1 |  |  |  |
| White and Mexican | 1 | 1 |  |  |  |
| White British | 71 | 71 |  |  |  |
| Did not answer | 3 | 3 |  |  |  |
| **Total** | **100** | **100%** |  |  |  |

## **fNIRS: Array and Cap Placement, Pre-processing, Channel Locations, Analyses, Head Modelling and Image Reconstruction**

### **Visualisations of the fNIRS Array**

A channel map is provided in **Supplementary Table 1** below that displays the position and channel numbers of the fNIRS probe associated with this research. Note that this is identical to that used at 10-months (as reported in Fiske et al., 2022) and 16-months (Fiske et al., 2024). The black lines (representing channels) were manually added to this figure for visualisation purposes, and so are not scaled accurately. See **Section 2.4** below for the anatomical labels of the channels in the fNIRS probe. **Supplementary Figure 2** provides a sensitivity heat map for the fNIRS probe (Fiske et al., 2022), and **Supplementary Figure 3** displays a visualisation of the fNIRS probe projected onto the cortex, taken from the 16-month paper (Fiske et al., 2024).

**Supplementary Figure 1.** Channel Map of the fNIRS probe.

| 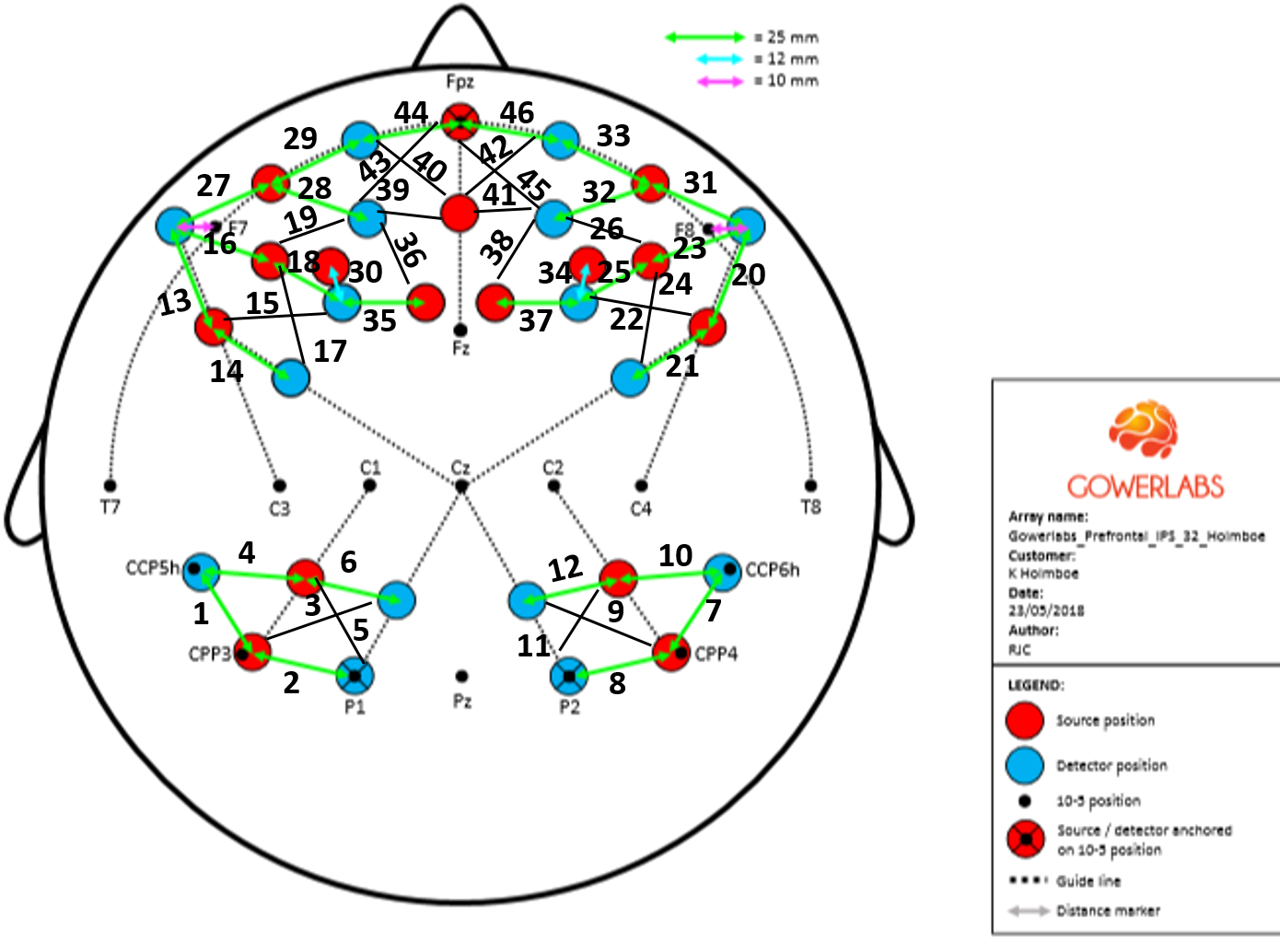 |
| --- |
| *Note.* Channels 1 – 12 overlay regions of the bilateral intraparietal sulcus (parietal cortex) and Channels 13 – 46 overlay regions of the bilateral prefrontal cortex. The source-detector separation between each nearest neighbour pair is 25mm, which is sufficient to sample cortical activity in infants (Lloyd-Fox et al., 2010; Taga et al., 2007). |

**Supplementary Figure 2.** fNIRS Array Sensitivity Map.

| 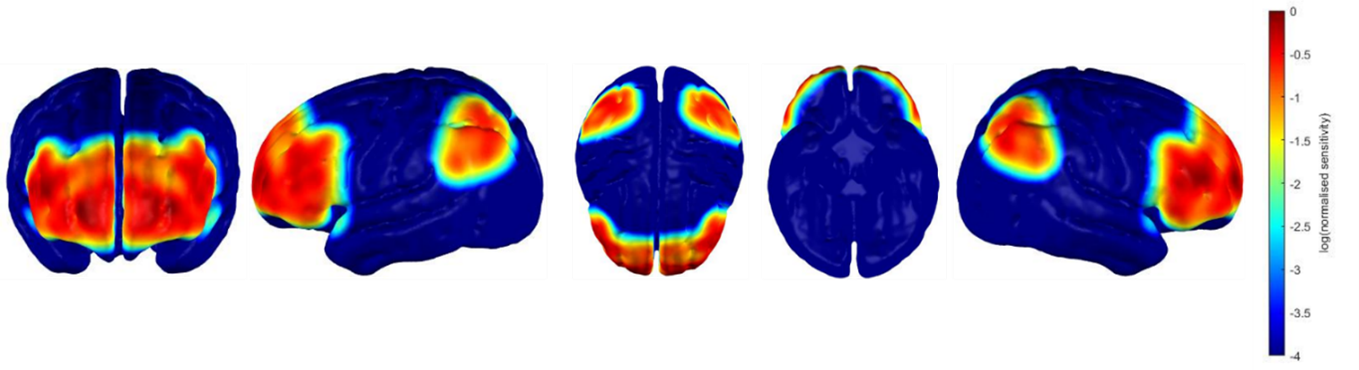 |
| --- |
| *Note*. This sensitivity heat map was generated on a 12-month-old infant head model (Shi et al., 2011) using the optode positions associated with this probe. These maps are scaled to the maximum sensitivity value in the grey matter mesh and are displayed on a log normalised scale. From left to right: frontal view, left view, superior view, inferior view, and right view of the sensitivity of channels in this array. Figure and figure caption taken from Fiske et al. (2022), *NeuroImage*. |

**Supplementary Figure 3**. fNIRS probe channel positions.

| 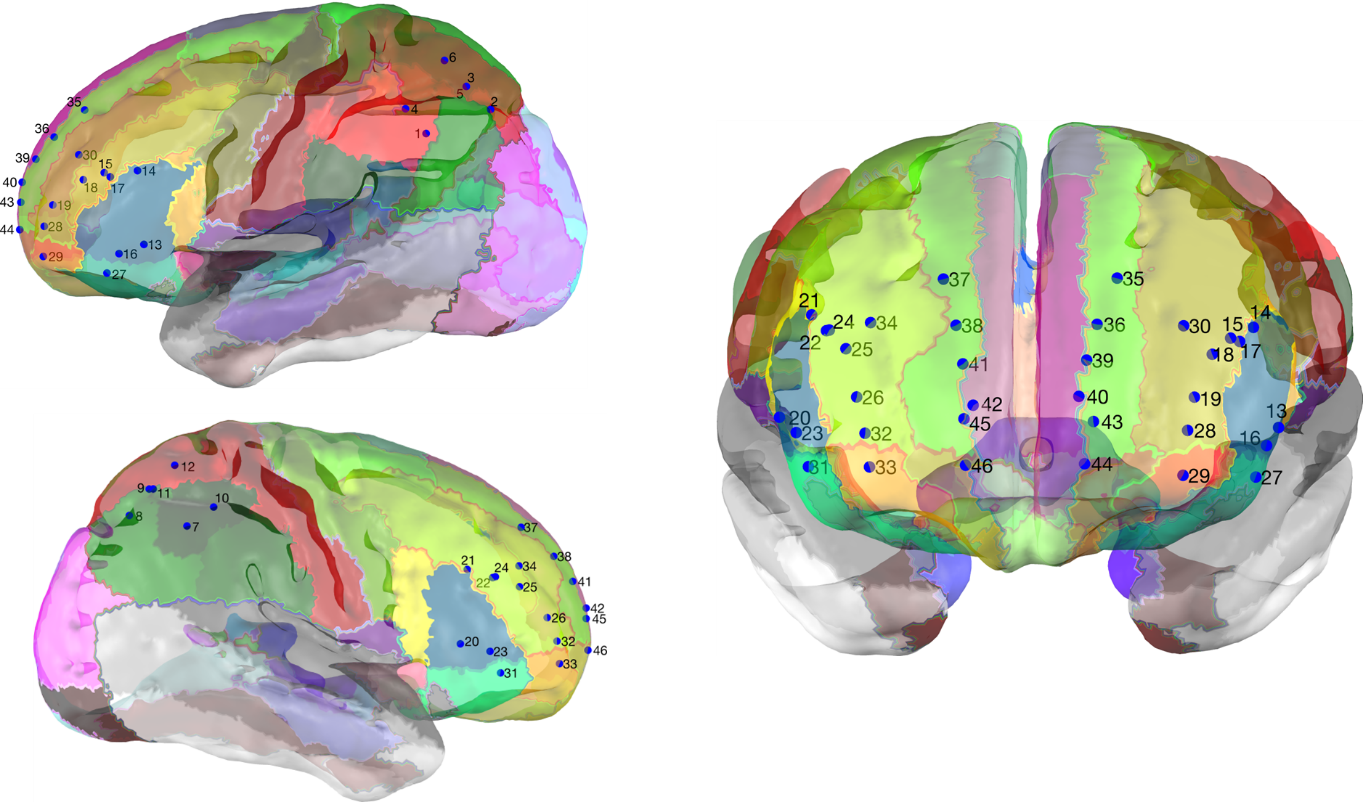 |
| --- |
| *Note.* Blue points represent channel positions on the cortex and numbers refer to the channel numbers. See Supplementary Materials 2.1 for a 2D channel map, and Supplementary Materials 2.4 for a table providing the anatomical location of each channel. The fNIRS probe covers the bilateral prefrontal cortex and the bilateral area around the intraparietal sulcus. Head model is based on averaged structural MRI data of a 12-month-old cohort of infants (Shi et al., 2011) and was scaled to the group mean head circumference measurement of participants in this study with useable fNIRS data. The positions of sources and detectors were registered virtually to the scalp surface of the head model using the HomER2 spring relaxation mechanism (Aasted et al., 2015). MATLAB figures of the channel positions on the cortex are available to view [here](https://www.dropbox.com/scl/fi/7xf7yapsvzwykfqit347t/ChannelNumber.fig?rlkey=wquic0jieo5n2pztephgb0lxb&st=i53i6osl&dl=0). Figure and figure caption taken from Fiske et al. (2024), *Imaging Neuroscience*. |

### **fNIRS Cap Placement**

Experimenters were trained to place the fNIRS cap on the participant’s head in a specific way in attempt to standardise the cap placement: the front of the cap sat just above the eyebrows, and the central optode (anchored to FpZ) was positioned centrally between the eyebrows (see Figure 1). As in our previous work, the validity of the headgear placement was assessed by two independent coders who viewed photographs of the cap placement and rated the headgear placement using a simple coding [scheme](https://osf.io/cu6wp). Different sized caps were used at each assessment point to best fit the head circumference of each participant.

### **fNIRS Pre-processing in HomER2**

See **Supplementary Table 3** for the processing functions and parameters (for HomER2 (Huppert et al., 2009)) used in this study.

**Supplementary Table 3.** HomER2 processing stream parameters.

| hmrIntensity2OD |  |  |
| --- | --- | --- |
| enPruneChannels | dRange | 1e-03 1e+03 |
|  | SNRthresh | 2 |
|  | SDrange | 0.0 45.0 |
|  | reset | 0 |
| hmrMotionArtifactByChannel | tMotion | 1.0 |
|  | tMask | 1.0 |
|  | STDEVthresh | 15.5 |
|  | AMPthresh | 0.40 |
| hmrMotionCorrectSpline | p | 0.99 |
|  | turnon | 1 |
| hmrMotionCorrectWavelet | iqr | 1.5 |
|  | turn_on | 1 |
| hmrMotionArtifactByChannel | tMotion | 1.0 |
|  | tMask | 1.0 |
|  | STDEVthresh | 15.5 |
|  | AMPthresh | 0.40 |
| hmrBandpassFilt | hpf | 0.010 |
|  | lpf | 0.80 |
| hmrOD2Conc | ppf | 5.2 4.8 |
| enStimIncData_varagin | 1 | 0.0 0.0 |
| hmrBlockAvg | trange | -2.0 20.0 |

### **Head Modelling, Channel Localisation and Image Reconstruction**

The below section of text has been taken from **Section 2.6.5.** of our previous publication (Fiske et al., 2022).

To allow us to visualise the data on an age-appropriate head template, a model of the infant head was produced from averaged structural MRI data of a 12-month-old cohort (Shi et al., 2011). Group-level tissue masks were combined to produce a mask of the spatial distribution of the cerebral tissues (white matter, grey matter, and cerebrospinal fluid). The inner skull border was delineated by the outside boundary of the cerebral tissue mask, while the scalp surface was defined using the Betsurf procedure (Jenkinson et al., 2005) where the group-level T1-weighted MRI template was used as an input. All voxels situated between the inner skull border and the scalp surface were assigned to be extra-cerebral tissue; this is a combined label for scalp and skull and is commonly done in infant head models due to the difficulty in discerning these two tissues in infant MRI data (Brigadoi et al., 2019; Collins-Jones et al., 2021; Frijia et al., 2021). The resulting four-layer tissue mask (consisting of white matter, grey matter, cerebrospinal fluid and extra-cerebral tissue) was converted to a tetrahedral volume mesh and a grey matter surface mesh using the iso2mesh package; Fang & Boas, 2009, see iso2mesh.sourceforge.net).

As demonstrated by Collins-Jones et al. (2021), assuming a constant head size and array position is a valid approach for an image reconstruction approach using infant fNIRS data. The head model was scaled to the group mean head circumference measurement of the 59 infants in this study with useable fNIRS data. The positions of sources and detectors were registered virtually to the scalp surface of the head model using the Homer2 spring relaxation mechanism (Aasted et al., 2015). To model the transport of near-infrared light through the head model, TOAST++ ((Schweiger & Arridge, 2014), see http://toastplusplus.org) was employed to produce a forward model for each wavelength. Using the group-level block-averaged optical density data, the forward model was then used to reconstruct a time-series of images of HbO2 and HHb concentration changes for each condition. Image reconstruction was constrained to the grey matter nodes of the volume mesh, as per previous topographic approaches (Boas et al., 2004; Boas & Dale, 2005). The resulting reconstructed images were mapped to the grey matter surface mesh. Data preparation, meshing, forward modelling, and reconstruction were facilitated by the DOT-HUB Toolbox (www.github.com/DOT-HUB).

The cortical positions for channels showing significant experimental effects (reported in Section 3.2 of Fiske et al., 2022) were determined using the forward model. For each channel, the sensitivity values from the forward model mapped to the grey matter surface were used to compute a weighted average of grey matter node positions; the nearest grey matter node to the weighted average position was determined. Using the infant automated anatomical labelling (AAL) atlas presented by Shi et al. (2011), the anatomical label of the nearest grey matter node was determined and was assigned as the cortical label of the channel.

***From the pre-registration associated with the 16-month paper (***[***https://osf.io/hpb4s***](https://osf.io/hpb4s)***):***

Note: A small change to the head modelling and channel localisation procedure was made for the 16-month dataset, such that there are some minor differences between the procedure used to localise channels at 10- and 16-months; see section ‘Prior knowledge’ for full details. This means that the label for Channel 32 (which is positioned on the border between the ‘middle frontal gyrus’ and the ‘middle frontal gyrus, orbital’ regions, according to the infant automated anatomical labelling atlas; Shi et al. (2011) has changed from the ‘middle frontal gyrus, orbital’ (as used in the 10-month paper; Fiske et al., 2022) to the ‘middle frontal gyrus’ label. Nevertheless, the position of this channel is closer to the right orbital region than the previously identified channels of interest in the right dorsolateral region (Channels 25 and 26; Fiske et al., 2022). Therefore, for the purposes of the analyses planned in this pre-registration, we consider Channel 32 to be part of the OFC and will use this label throughout our pre-registration. This also retains consistency in labelling between the current pre-registration and Fiske et al. (2022). MATLAB figures of the channel positions on the cortex at both 10- and 16-months are available to view [here](https://www.dropbox.com/sh/pe6qj8u67gq1hif/AADehL3tbJEGbyMZo5Q0tncwa?dl=0).

### **Anatomical Labels of Channels**

This work was completed by Dr Liam Collins-Jones for the [pre-registered](https://osf.io/swe2j) study associated with these Supplementary Materials. **Supplementary Table 4** below provides the anatomical label for each channel in the fNIRS probe used in the current study. The only changes in anatomical labels from 16-months to 3½ years were in Channel 9 (angular gyrus at 16-months to inferior parietal gyrus at 3½ years) and Channel 39 (superior frontal gyrus, dorsolateral at 16-months to superior frontal gyrus, medial at 3½ years).

**Supplementary Table 4.** Anatomical labels of channels in the fNIRS probe at 16-months.

| Channel | Hemisphere | Cortical Region |
| --- | --- | --- |
| 1 | Left | 'Inferior parietal gyrus (P2)' |
| 2 | Left | 'Inferior parietal gyrus (P2)' |
| 3 | Left | 'Superior parietal gyrus (P1)' |
| 4 | Left | 'Inferior parietal gyrus (P2)' |
| 5 | Left | 'Superior parietal gyrus (P1)' |
| 6 | Left | 'Superior parietal gyrus (P1)' |
| 7 | Right | 'Inferior parietal gyrus (P2)' |
| 8 | Right | 'Angular gyrus (AG)' |
| 9 | Right | ‘Inferior parietal gyrus (P2)' |
| 10 | Right | 'Inferior parietal gyrus (P2)' |
| 11 | Right | 'Superior parietal gyrus (P1)' |
| 12 | Right | 'Superior parietal gyrus (P1)' |
| 13 | Left | 'Inferior frontal gyrus, triangular (F3T)' |
| 14 | Left | 'Inferior frontal gyrus, triangular (F3T)' |
| 15 | Left | 'Middle frontal gyrus (F2)' |
| 16 | Left | 'Inferior frontal gyrus, triangular (F3T)' |
| 17 | Left | 'Middle frontal gyrus (F2)' |
| 18 | Left | 'Middle frontal gyrus (F2)' |
| 19 | Left | 'Middle frontal gyrus (F2)' |
| 20 | Right | 'Inferior frontal gyrus, triangular (F3T)' |
| 21 | Right | 'Middle frontal gyrus (F2)' |
| 22 | Right | 'Middle frontal gyrus (F2)' |
| 23 | Right | Inferior frontal gyrus, triangular (F3T)' |
| 24 | Right | 'Middle frontal gyrus (F2)' |
| 25 | Right | 'Middle frontal gyrus (F2)' |
| 26 | Right | 'Middle frontal gyrus (F2)' |
| 27 | Left | 'Inferior frontal gyrus, orbital (F3O)' |
| 28 | Left | 'Middle frontal gyrus (F2)' |
| 29 | Left | 'Middle frontal gyrus, orbital (F2O)' |
| 30 | Left | 'Middle frontal gyrus (F2)' |
| 31 | Right | 'Inferior frontal gyrus, orbital (F3O)' |
| 32 | Right | 'Middle frontal gyrus (F2)' |
| 33 | Right | 'Middle frontal gyrus, orbital (F2O)' |
| 34 | Right | 'Middle frontal gyrus (F2)' |
| 35 | Left | 'Superior frontal gyrus, dorsolateral (F1)' |
| 36 | Left | 'Superior frontal gyrus, dorsolateral (F1)' |
| 37 | Right | 'Superior frontal gyrus, dorsolateral (F1)' |
| 38 | Right | 'Superior frontal gyrus, dorsolateral (F1)' |
| 39 | Left | 'Superior frontal gyrus, medial (F1M)' |
| 40 | Left | 'Superior frontal gyrus, medial (F1M)' |
| 41 | Right | 'Superior frontal gyrus, dorsolateral (F1)' |
| 42 | Right | 'Superior frontal gyrus, medial (F1M)' |
| 43 | Left | 'Superior frontal gyrus, dorsolateral (F1)' |
| 44 | Left | 'Superior frontal gyrus, medial orbital (F1MO)' |
| 45 | Right | 'Superior frontal gyrus, dorsolateral (F1)' |
| 46 | Right | 'Superior frontal gyrus, orbital (F1O)' |
| *Note.* The anatomical labels for each channel were created using the infant automated anatomical labelling (AAL) atlas presented by (Shi et al., 2011). Work conducted by Dr Liam Collins-Jones. | | |

### **Overview of the fNIRS Preprocessing and Analysis Pipeline**

The purpose of this section is to provide a full step-by-step outline of the fNIRS preprocessing and analysis pipeline used in the current study. All of the below steps are also outlined in the [pre-registration](https://osf.io/swe2j). A flow chart that summarises the key processes is provided in **Supplementary Figure 4** for visualisation purposes.

1. **Preprocessing in HomER2**

First, the channel-level fNIRS data for each participant were pre-processed in HomER2. The full processing pipeline is provided in **Supplementary Table 3** above, but is summarised below:

- Raw intensity data were converted to optical density data
- Channels with an optical density that was too high or too low (1e – 03, 1e + 03) were excluded from further processing and analyses
- Motion artifacts were identified at the channel level and corrected using Spline and Wavelet
- A band pass filter was applied at the channel level (high pass filter: 0.010, low pass filter: 0.80) to filter out physiological noise or other artifacts
- Optical density data were converted to haemoglobin concentration data
- Manually excluded events were removed from the data
- Data for each block-type (control, experimental) were averaged across a period of 22 seconds, which contained 2s of the preceding baseline and 20 seconds of the block time course.

1. **Primary Statistical Analyses – Channel Level**

Next, we conducted our primary statistical analyses on the pre-processed fNIRS data at the channel-level. As outlined in our pre-registration, we had several planned analyses routes depending on whether our pre-registered hypothesis was confirmed. Our hypothesis was that there would be a significant block-type effect in nine channels covering the left superior parietal cortex (Channel 6), right inferior parietal cortex (Channel 8), right IFG (Channels 20 and 23), right DLPFC (Channel 26), left DLPFC (Channel 28), left OFC (Channel 29), right IFG, orbital (Channel 31) and right OFC (Channel 33), such that activation was greater when inhibition was required in experimental blocks, compared to in control blocks where inhibition was not required. This hypothesis was based on the results of our previous studies with the same participants when they were 10-month-old (Fiske et al., 2022) and 16-months old (Fiske et al., 2024). The first step in our analysis was to conduct repeated measures ANOVAs on the nine pre-registered channels specified above. The next step would depend on the results of the repeated measures ANOVAs. See **Figure 3** of the main manuscript for an illustration of our analysis approach.

- **Route 1: All nine pre-registered channels showed a significant block-type effect**
  - Confirmatory analyses
  - We will conduct paired t-tests on all nine channels to examine the time course of the block-type effect
  - We will conduct correlational analyses to examine the potential association between activation in all nine channels and individual differences in task performance.
- **Route 2: Some of the pre-registered channels showed a significant block-type effect**
  - Confirmatory analyses
  - We will conduct paired t-tests on only the channels that showed significant block-type effects to examine the time course of the block-type effect
  - We will conduct correlational analyses to examine the potential association between activation in the channels showing significant block-type effects and individual differences in task performance.
- **Route 3: None of the pre-registered channels showed a significant block-type effect**
  - No further analyses would be conducted on these channels

Following this, we will then conduct exploratory analyses on the remaining channels in the probe that were not pre-registered.

- **Route 4: All remaining channels**
  - Exploratory analyses
  - We will conduct repeated measures ANOVAs to examine which of the remaining channels were showing significant block-type effects
  - We will conduct paired t-tests on all channels showing significant block-type effects to examine the time course of the block type effect
  - We will conduct correlational analyses to examine the potential association between activation in all significant channels and individual differences in task performance.

1. **Head Modelling and Channel Localisation**

In order to localise the channels in our fNIRS probe to anatomical regions on the head (and so provide an anatomical label for each channel number), we followed the procedure outlined in **Section 2.4.** of the current Supplementary Materials. This approach involved using a forward model and an age-relevant head model to provide anatomic labels for each channel position. A list of anatomical labels can be found in **Supplementary Table 4**.

1. **Secondary Analyses for Image Reconstruction – Image Space**

In order to visualise the significant block-type effects identified in our primary analyses, we conducted a secondary analysis to produce an image reconstruction of our results. This approach is described in detail in **Section 2.4.** of the current Supplementary Materials. In brief, for each participant the block-averaged changes in optical density data^*^ are used (in combination with the forward model computed to determine channel location) to reconstruct a concentration change image (one for HbO_2_, one for HHb) for each 5s time window during the block-averaged 20s time window. Then, the values at each node across participants during these 5s time windows are compared to a 2s baseline using a two-sample *t*-test to produce the image (**Figure 5** of the main manuscript).

^*^ Note: Since these are linear processes, the block-averaged changes in optical density we have obtained would be exactly the same as though we had directly computed them without converting to and from concentration changes.

**Supplementary Figure 4.** Flow Chart of fNIRS Analytic Pipeline.

| 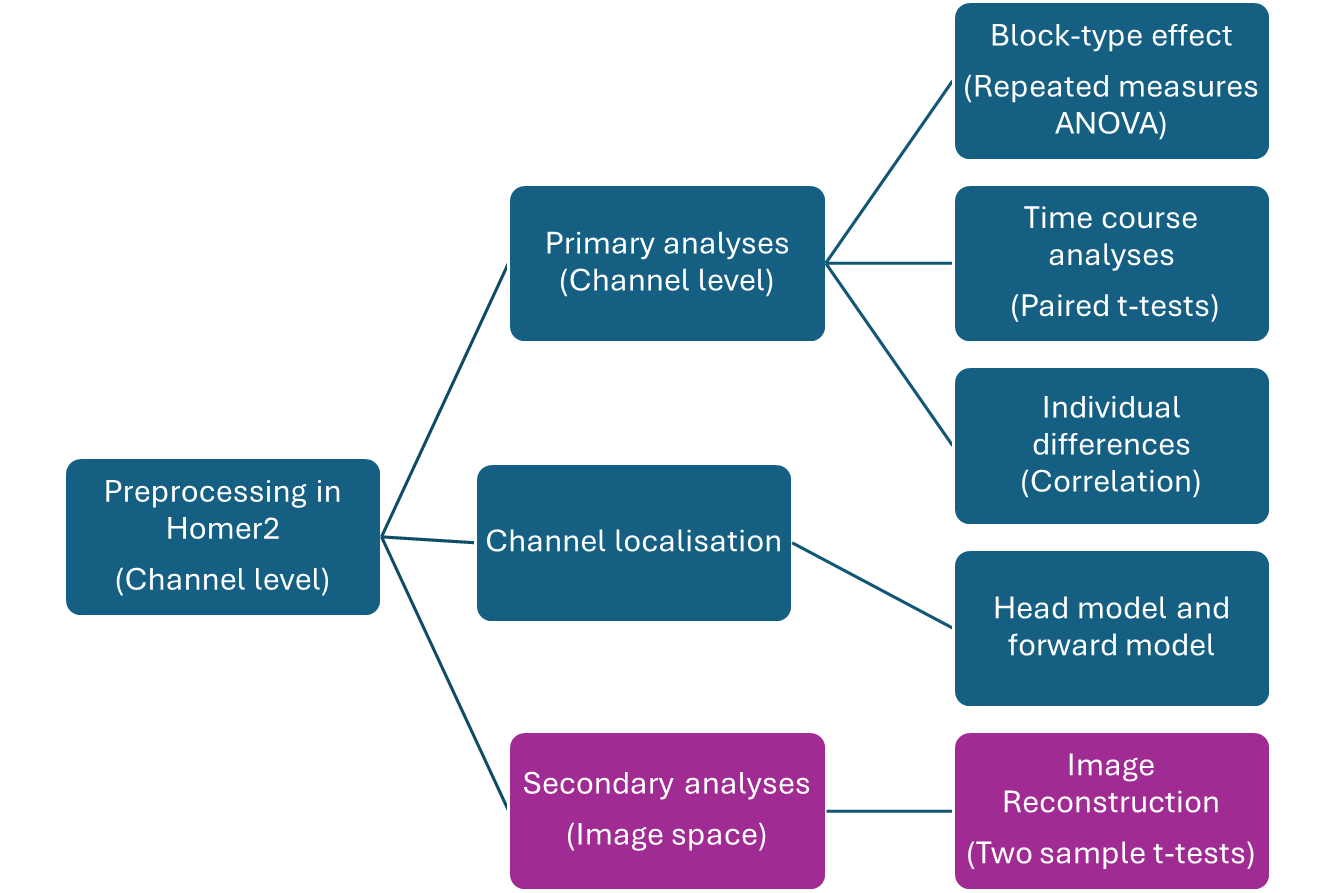 |
| --- |
| *Note.* This figure displays the analytic pipelines followed for the fNIRS data. Blue boxes denote channel-level analyses and purple boxes show analyses conducted in image space. |

## **Pre-registered hypotheses, analysis plans and variables**

### **Pre-registered hypotheses and analysis plans**

Our pre-registered hypotheses and analysis plans for our behavioural data from the ECITT are reported below in **Supplementary Table 5** (3½ years) and **Supplementary Table 6** (longitudinal). The following behavioural indices will be generated from the ECITT: mean prepotent accuracy, mean inhibitory accuracy, median prepotent response time (correct trials only), median inhibitory response time (correct trials only), accuracy inhibitory score. See **Supplementary Materials 3.2** below for further information about the variables used in this study. The pre-registered hypotheses and analysis plans for our fNIRS data can be found in **Supplementary Table 7** (3½ years) **Supplementary Table 8** (longitudinal). The pre-registration on OSF can be found [here](https://osf.io/swe2j).

**Supplementary Table 5.** Pre-registered Behavioural Hypotheses and Analysis Plans.

| **Behavioural Data (ECITT)** | **Pre-registered Hypothesis** | **Pre-registered Analysis Plan** |
| --- | --- | --- |
| **Block-type Effects**  *Based on previous findings of Fiske et al. (2022, 2024),* Hendry et al. (2021) *and* Holmboe et al. (2021) | **H1:** Participants will be significantly more accurate on prepotent than inhibitory trials.  **H2:** Participants will respond significantly faster on correct prepotent than correct inhibitory trials. | **H1:** Paired *t*-test with the mean accuracy variables (inhibitory, prepotent)  **H2:** Paired *t*-test with the median response time variables (inhibitory, prepotent) |
| **Group Performance Differences**  *Based on previous findings of Fiske et al. (2022, 2024)*  *Participants were grouped depending on whether they contributed: both valid ECITT and fNIRS data (N = 61) or only valid ECITT data (N = 39).* | **H3a:** There will be no significant difference in accuracy between groups  **H3b:** Participants who contributed both valid ECITT and fNIRS data would have significantly faster median reaction time on both correct prepotent and inhibitory trials. | Two 2 × 2 mixed ANOVAs with trial-type as within-subjects factor (inhibitory, prepotent) and group (fNIRS data, no fNIRS data) as the between-subjects factor.  **H3a:** Mean accuracy data  **H3b:** Median response time data |
| **Between-Session Consistency**  *Based on previous findings of Fiske et al. (2022, 2024)* | **H4a:** Participants will be significantly more accurate on inhibitory trials in the blocked ECITT (Session 2) than in the behavioural ECITT (Session 1).  **H4b:** Participants will respond significantly faster on both trial types, on the blocked ECITT (Session 2) than on the behavioural ECITT (Session 1). | Two 2 × 2 repeated measures ANOVAs with trial type (prepotent, inhibitory) and session (behavioural ECITT in Session 1, blocked ECITT in Session 2) as within-subjects factors.  **H4a:** Mean accuracy data as DV  **H4b:** Median response time data as DV  Paired *t*-test with the accuracy inhibitory score as DV to investigate group-level stability in overall inhibitory performance across sessions. |
| **Test Re-Test Reliability**  *Based on previous findings of Fiske et al. (2022, 2024)* | **H5:** There will be a significant correlation of at least a medium effect size (*r* ≥ .3) in performance between sessions for all variables. | Five one-tailed correlational analyses will be conducted between data from both versions of the ECITT.  DVs: accuracy inhibitory score, mean prepotent accuracy, mean inhibitory accuracy, median prepotent response time, median inhibitory response time. |
| *Note.* DV = dependent variable. | | |

**Supplementary Table 6.** Pre-registered Longitudinal Behavioural Hypotheses and Analysis Plans.

| **Behavioural Data (ECITT)** | **Pre-registered Hypothesis** | **Pre-registered Analysis Plan** |
| --- | --- | --- |
| **Development from Infancy to Early Childhood**  *Based on previous findings of* Holmboe et al. (2021)  *[Reported in Supplementary Materials]* | **H6a:** Development from 10-months to 3½ years  There will be a significant main effect of trial type (greater accuracy on prepotent compared to inhibitory trials), a significant main effect of age (greater accuracy at 3½ years than at 10-months), and a significant interaction (larger improvement in inhibitory accuracy than in prepotent accuracy between 10 months and 3½ years).  Participants would be significantly more accurate on both trial types and would have significantly better response inhibition performance at 3½ years than at 10-months | **H6a:** 2 × 2 linear mixed model with trial type (prepotent, inhibitory) and age (10-months and 3½ years) as within-subjects factors.  Paired *t*-test with the accuracy inhibitory score as DV to investigate whether inhibitory performance is significantly different from 10-months to 3½ years. |
|  | **H6b:** Development from 16-months to 3½ years  *Accuracy*: There will be a significant main effect of trial type (greater accuracy on prepotent compared to inhibitory trials), a significant main effect of age (greater accuracy at 3½ years than at 16-months), and a significant interaction (larger improvement in inhibitory accuracy than in prepotent accuracy between 16-months and 3½ years).  *Response time*: There would be a significant main effect of trial type (faster responses on correct prepotent compared to on correct inhibitory trials), no significant main effect of age, and no significant interaction. There will be no significant difference in median reaction times on both trial types between 16-months and 3½ years. | **H6b:** Two separate 2 × 2 linear mixed models with trial type (prepotent, inhibitory) and age (16-months and 3½ years) as within-subjects factors   1. Mean accuracy data as DV 2. Median (correct) response time data as DV   Paired *t*-test with the accuracy inhibitory score as DV to investigate whether inhibitory performance is significantly different from 16-months to 3½ years. |
|  | **H7**: It was predicted that performance will not be significantly correlated at 10-months and 3½ years (H7a), or 16-months and 3½ years (H7b).  **H7b**: There will be no significant correlation between the median inhibitory reaction time on the blocked ECITT at 16-months and 3½ years. There will be a significant correlation between median prepotent reaction time at 16-months and 3½ years. | **H7**: Two separate correlational analyses between performance (accuracy inhibitory score) on the blocked ECITT at 10-months and 3½ years (H7a), or at 16-months and 3½ years (H7b).  **H7b:** Two further correlational analyses between reaction time (median prepotent, median inhibitory) at 16-months and 3½ years. |
| *Note.* DV = dependent variable.  **Supplementary Table 7.** Pre-registered fNIRS Hypotheses and Analysis Plans. | | |

| fNIRS Data | Pre-registered Hypothesis | Pre-registered Analysis Plan |
| --- | --- | --- |
| Identifying Channels Showing Significant Block-Type Effects  *Based on findings from the 16-month dataset (Fiske et al., 2024)*  *[Time course - reported in Supplementary Materials]* | H8a: The haemodynamic response in nine channels* overlying regions of the PFC and parietal cortex will be differentiated by block type (i.e., ‘experimental’ blocks requiring inhibition vs. ‘control’ blocks with no inhibitory requirement).  *Channel 6 (left superior parietal), Channel 8 (right inferior parietal), Channels 20 and 23 (right IFG), Channel 26 (right DLPFC), Channel 28 (left DLPFC), Channel 29 (left OFC), Channel 31 (right IFG, orbital), and Channel 33 (right OFC).  H8b: Exploratory – no specific hypothesis. | H8a (confirmatory): Data will be divided into five 4-second time bins (0 – 20 seconds of the block time course). Repeated measures ANOVAs will be conducted for each channel and each chromophore (HbO_2_ and HHb) with time bin (5 levels) and block type (2 levels) as within-subjects factors.  Channels showing a significant main effect of block type, or a significant time × block type interaction in either chromophore will be considered as showing a significant block type effect (i.e., haemoglobin concentration is differentiated by block type).  H8b (exploratory): Once H8a has been tested, exploratory repeated measures ANOVAs (as described above) will be conducted on the remaining channels in the probe to investigate whether any other channels are showing significant block type effects.  H8a and H8b (exploratory): If channels show significant block type effects, two-tailed paired t-tests will be conducted to investigate the time course of the significant effect. |
| Individual Differences (Brain-Behaviour)  *[Reported in Supplementary Materials]* | H9: Exploratory – no specific hypothesis.  Since findings from the 10-month dataset relating to potential associations between individual performance differences and inhibition-specific brain activation were weak and did not survive correction for multiple comparisons (Fiske et al., 2022), and there were no significant associations in the 16-month dataset (Fiske et al., 2024), no specific predictions were made about whether there would be significant brain-behaviour associations at 3½ years. | H9 (exploratory): Once channels displaying significant block type effects have been identified (see H8), two-tailed correlational analyses will be conducted to investigate whether individual differences in neural activation in these channels (across the time bins showing significant effects) are associated with individual differences in the accuracy inhibitory score. |
| *Note.*  IFG = inferior frontal gyrus, DLPFC = dorsolateral prefrontal cortex, OFC = orbital frontal cortex, HbO_2_ = oxygenated haemoglobin, HHb = deoxygenated haemoglobin. | | |

**Supplementary Table 8.** Pre-registered Longitudinal fNIRS Hypotheses and Analysis Plans.

| fNIRS Data | Pre-registered Hypothesis | Pre-registered Analysis Plan |
| --- | --- | --- |
| Neural Development from Infancy to Early Childhood | H10: Exploratory – no specific hypothesis. | H10 (exploratory): Once H8 has been tested:  Two separate exploratory linear mixed model analyses will be conducted on channels showing significant block type effects at the 10-month and/or 16-month assessment points, and any new channels identified at 3½ years. These analyses will be conducted to investigate the change in activation in each channel across assessment points.  Model 1: Change from 10-months to 3½ years  Model 2: Change from 16-months to 3½ years  DV: Haemoglobin difference measure  Fixed factor: Assessment Point  Random factor: Participants |
| *Note.* DV = dependent variable. | | |

### **Description of Variables**

The information provided below about the variables used in the current study has been taken from the pre-registration associated with this study (<https://osf.io/swe2j>).

**Measured variables**

Behavioural data (from the ECITT):

The measured variables in this task were trial-level accuracy (0 or 1) and reaction time (ms). Only valid trials (trials where the reaction time was > 300 ms and < 5000 ms) will be included in analyses. For reaction time analyses, only correct and valid trials will be included.

- Prepotent trial accuracy: infants’ accuracy on each valid* prepotent trial. Scored as 0 (incorrect) or 1 (correct).
- Inhibitory trial accuracy: infants’ accuracy on each valid* inhibitory trial. Scored as 0 (incorrect) or 1 (correct).
- Prepotent trial reaction time (ms): calculated by the ECITT software from the onset of the trial until a touch is detected in the target response area (or as corrected during video coding* where the response was not detected by the iPad). Note that only valid and correct prepotent trials will be included in the reaction time analyses.
- Inhibitory trial reaction time (ms): calculated by the ECITT software from the onset of the trial until a touch is detected in the target response area.

fNIRS data:

The fNIRS hardware (Gowerlabs NTS system) measures a raw intensity signal (in two wavelengths: 780 and 850 nm) that then undergoes several transformations during pre-processing (e.g., conversion to optical density data and then to haemoglobin concentration data) to provide the measure of interest for our analysis: the average HbO_2_ and HHb concentration per individual, per channel, per time bin.

- An average HbO_2_ concentration variable will be generated for each participant with valid fNIRS data, for each time bin, for each channel (N ~46; although actual number may vary following exclusions).
- An average HHb concentration variable will be generated for each participant with valid fNIRS data, for each time bin, for each channel (N ~46; although actual number may vary following exclusions).

**Behavioural indices (from the ECITT):**

We will generate the same behavioural index of accuracy performance on the ECITT as described and used in our previous work (Fiske et al., 2022, 2024). This will be generated for both the behavioural and blocked versions of the ECITT.

- Mean prepotent accuracy: mean accuracy across all valid prepotent trials. Scored from 0 (all incorrect) to 1 (all correct).
- Mean inhibitory accuracy: mean accuracy across all valid inhibitory trials. Scored from 0 (all incorrect) to 1 (all correct).
- Accuracy inhibitory score: used as an index of response inhibition performance. A larger accuracy inhibitory score is indicative of better response inhibition ability. In cases where mean prepotent accuracy > mean inhibitory accuracy, the accuracy inhibitory score will be calculated using the following formula: (1 – (mean prepotent accuracy – mean inhibitory accuracy)/mean prepotent accuracy). In cases where mean inhibitory accuracy > mean prepotent accuracy, the accuracy inhibitory score will be calculated using a different formula: (1 – (mean prepotent accuracy – mean inhibitory accuracy) × mean prepotent accuracy). Based on our previous work, we expect that only a small number of participants will have a higher mean inhibitory accuracy score than mean prepotent accuracy score.
- Median prepotent reaction time: The median reaction time on valid correct prepotent trials (for each individual). When conducting group level analyses, the mean of the individual medians will be used.
- - Median inhibitory reaction time: The median reaction time on valid correct inhibitory trials (for each individual). When conducting group level analyses, the mean of the individual medians will be used.

Note that reaction time data was not used at 10-months (Fiske et al., 2022). For discussion of why we do not consider reaction time data collected from 10-month-old infants to be valid, see Lui et al. (2021) and Hendry et al. (2021).

## **Parametric Test Assumptions**

Each behavioural and fNIRS-related variable was tested for normality by examining the results of the Shapiro-Wilk test and the skewness (z-score) of the data; the results and conclusions of these tests are reported in **Supplementary Table 9** below. The significance value of the Shapiro-Wilk test informs whether the data are normally distributed; if *p* > .005, this suggests that the data are normally distributed, but if p < .005, this suggests that the data may deviate from a normal distribution. According to Kim (2013), for samples with more than 50 and less than 300 participants, a z-scored skew > 3.29 would suggest the sample distribution is not normal. This criterion has been applied to the behavioural variables used in this study when determining normality. In cases where variables are not normally distributed and are skewed beyond an acceptable level, non-parametric tests will be conducted alongside parametric tests to check for convergence.

Similarly, each fNIRS variable was tested for normality before the repeated measures ANOVAs were conducted. The data meet the assumptions that the dependent variable (haemoglobin concentration) must be on a continuous scale and that the within-groups variable (condition) must have two or more groups (control, experimental). Due to the extremely high number of variables (N = 1933), it was not possible to test the dependent variable (haemoglobin concentration data) for normality. The dependent variables are unlikely to be normally distributed due to the nature of the physiological data and the sheer number of variables. However, the repeated measures ANOVA is robust against violations of the normality assumption and so the risk of Type I error remains low. To control for the possibility of false positives, the Benjamini-Hochberg procedure for controlling the false discovery rate has been applied to these analyses (Benjamini & Hochberg, 1995). The sphericity of the data was tested using Mauchly’s test of sphericity and data from all but one channel (Channel 15) did not meet the sphericity assumption. Therefore, the Greenhouse-Geisser degrees of freedom and p-values were reported to account for the lack of sphericity in all channels.

**Supplementary Table 9.** Normality Tests for the 3½ year Dataset.

|  | Variable | *N* | Shapiro-Wilk (*p*) | Skew (z) | Conclusion |
| --- | --- | --- | --- | --- | --- |
| Behavioural ECITT  (Session 1) | Mean inhibitory accuracy | 113 | <.001 | -3.49 | **Not normally distributed, skew not acceptable** |
|  | Mean prepotent accuracy | 113 | <.001 | -7.79 | **Not normally distributed, skew not acceptable** |
|  | Accuracy inhibitory score | 113 | <.001 | -3.93 | **Not normally distributed, skew not acceptable** |
|  | Median inhibitory reaction time | 113 | <.001 | 6.77 | **Not normally distributed, skew not acceptable** |
|  | Median prepotent reaction time | 113 | <.001 | 5.86 | **Not normally distributed, skew not acceptable** |
| Blocked ECITT (Session 2) | Mean inhibitory accuracy | 100 | <.001 | -3.22 | Not normally distributed, acceptable skew |
|  | Mean prepotent accuracy | 100 | <.001 | -33.54 | **Not normally distributed, skew not acceptable** |
|  | Accuracy inhibitory score | 100 | <.001 | -3.41 | **Not normally distributed, skew not acceptable** |
|  | Median inhibitory reaction time | 100 | <.001 | 6.16 | **Not normally distributed, skew not acceptable** |
|  | Median prepotent reaction time | 100 | <.001 | 4.62 | **Not normally distributed, skew not acceptable** |
| Individual Differences | Channel 7 HHb Difference Bin 5 | 36 | .236 | 1.16 | Normally distributed, acceptable skew |
|  | Channel 8 HHb Difference Bins 4 - 5 | 33 | <.001 | 3.98 | **Not normally distributed, skew not acceptable** |
|  | Channel 18 HbO_2_ Difference Bins 3 - 4 | 50 | <.001 | -9.27 | **Not normally distributed, skew not acceptable** |
|  | Channel 29 HHb Difference Bin 2 | 57 | <.001 | -4.40 | **Not normally distributed, skew not acceptable** |
|  | Channel 31 HbO_2_ Difference Bins 1 - 2 | 52 | .217 | -1.20 | Normally distributed, acceptable skew |
|  | Channel 31 HHb Difference Bins 3 - 5 | 52 | .501 | 1.50 | Normally distributed, acceptable skew |
|  | Channel 43 HHb Difference Bins 1 – 3 | 41 | .924 | 0.31 | Normally distributed, acceptable skew |
|  | Channel 45 HbO_2_ Difference Bins 1 – 2 | 47 | .704 | -0.42 | Normally distributed, acceptable skew |
|  | | | | | |

## **Additional Results**

### **Estimated Marginal Means for Linear Mixed Models**

**Behavioural Development from Infancy to Early Childhood:**

To investigate whether overall accuracy on the ECITT improved from 10 months to 3½ years (H6a: Model 1), and from 16-months to 3½ years (H6b: Model 2), two separate 2 x 2 linear mixed models (restricted maximum likelihood estimation) were conducted with trial type and age as within-subjects factors. Results are reported in the manuscript but see **Supplementary Table 10** for estimated marginal means.

| Model 1:  (10-months and 3½ years) | | Estimated marginal mean | Standard error | Degrees of Freedom | Confidence interval |
| --- | --- | --- | --- | --- | --- |
| Trial Type | Inhibitory | .680 | .016 | 217.670 | .647, .712 |
|  | Prepotent | .956 | .004 | 182.178 | .948, .965 |
| Age | 10-months | .735 | .014 | 145.171 | .709, .762 |
|  | 3½ years | .901 | .010 | 106.763 | .880, .921 |
| Interaction | Inhibitory 10-months | .549 | .026 | 124 | .497, .601 |
|  | Prepotent 10-months | .922 | .008 | 124 | .907, .937 |
|  | Inhibitory 3½ years | .810 | .020 | 99 | .771, .850 |
|  | Prepotent 3½ years | .991 | .004 | 99 | .983, .999 |
| Model 2:  (16-months and 3½ years) | | **Estimated marginal mean** | **Standard error** | **Degrees of Freedom** | **Confidence interval** |
| Trial Type | Inhibitory | .673 | .021 | 126.793 | .633, .714 |
|  | Prepotent | .957 | .005 | 106.103 | .947, .968 |
| Age | 16-months | .730 | .019 | 91.636 | .693, .767 |
|  | 3½ years | .901 | .010 | 106.763 | .880, .921 |
| Interaction | Inhibitory 16-months | .536 | .039 | 80 | .465, .608 |
|  | Prepotent 16-months | .924 | .010 | 80 | .905, .943 |
|  | Inhibitory 3½ years | .810 | .020 | 99 | .771, .850 |
|  | Prepotent 3½ years | .991 | .004 | 99 | .983, .999 |

**Supplementary Table 10.** Estimated Marginal Means: Accuracy Improvement from Infancy to Early Childhood

To investigate whether overall reaction time on the ECITT improved from 16-months to 3½ years (H6b: Model 3), a 2 × 2 linear mixed model (restricted maximum likelihood estimation) was conducted with trial type and age as within-subjects factors. Results are reported in the manuscript and see **Supplementary Table 11** for estimated marginal means.

| Reaction Time (ms) | | Estimated marginal mean | Standard error | Degrees of Freedom | Confidence interval |
| --- | --- | --- | --- | --- | --- |
| Trial Type | Inhibitory | 1421 | 29 | 140.990 | 1364, 1479 |
|  | Prepotent | 1152 | 19 | 174.997 | 1116, 1189 |
| Age | 16-months | 1367 | 27 | 114.063 | 1314, 1420 |
|  | 3½ years | 1207 | 22 | 193.271 | 1164, 1250 |
| Interaction | Inhibitory 16-months | 1454 | 48 | 76 | 1358, 1549 |
|  | Prepotent 16-months | 1280 | 25 | 76 | 1231, 1330 |
|  | Inhibitory 3½ years | 1389 | 33 | 99 | 1324, 1455 |
|  | Prepotent 3½ years | 1024 | 28 | 99 | 969, 1080 |

**Supplementary Table 11.** Estimated Marginal Means: Reaction Time Improvement from Infancy to Early Childhood

### **Longitudinal Associations, Between-Session Consistency, Test Retest Reliability and Group Differences**

- - 1. **Longitudinal Associations:**

No significant associations were found between performance on the ECITT in infancy and early childhood (**Supplementary Table 12)**.

**Supplementary Table 12.** Performance on the ECITT: Longitudinal Associations.

|  | Statistic |
| --- | --- |
| Accuracy Inhibitory Score (10-months and 3½ years) | *r* (76) = -.069, *p* = .274, *CI* = -.290, .167 |
| Accuracy Inhibitory Score (16-months and 3½ years) | *r* (40) = -.238, *p* = .067, *CI* = -.482, .064 |
| Median Prepotent RT (16-months and 3½ years) | *r* (40) = .224, *p* = .085, *CI* = -.187, .538 |
| Median Inhibitory RT (16-months and 3½ years) | *r* (40) = .108, *p* = .256, *CI* = -.110, .402 |

- - 1. **Between-Session Consistency**

Accuracy: As hypothesised (H4), results of the 2 × 2 mixed ANOVA demonstrated a significant main effect of session, whereby participants were significantly more accurate in Session 2 (blocked ECITT) than in Session 1 (behavioural ECITT): *F* (1, 95) = 5.812, *p* = .025, ηp^2^ = .058, Cohen’s *f* = 0.22. The trial type × session interaction was not significant: *F* (1, 95) = .429, *p* = .514, ηp^2^ = .004, Cohen’s *f* = .000. Estimated marginal means are reported in **Supplementary Table 13**. Contrary to predictions, there was no significant difference between the accuracy inhibitory score in Session 1 and Session 2: *t* (95) = -.885, *p* = .189, Cohen’s *d* = -.090.

Reaction Time: As hypothesised (H4), there was a significant main effect of session whereby participants responded significantly faster in Session 2 than in Session 1: *F* (1, 95) = 137.172, *p* < .001, ηp^2^ = .591, Cohen’s *f* = 1.18. There was also a significant type × session interaction, whereby the difference in reaction time between trial types (faster on prepotent trials than on inhibitory trials) was larger in Session 2 than in Session 1: *F* (1, 95) = 9.796, *p* = .002, ηp^2^ = .093, Cohen’s *f* = 0.30. Estimated marginal means are reported in **Supplementary Table 13**.

**Supplementary Table 13.** Estimated marginal means for mixed ANOVAs (Group-Level Between Session Consistency).

| Accuracy | | Estimated marginal mean | Standard error | Confidence interval |
| --- | --- | --- | --- | --- |
| Trial type | Inhibitory | .806 | .015 | .776, .837 |
|  | Prepotent | .983 | .003 | .977, .988 |
| Session | 1 | .883 | .009 | .865, .901 |
|  | 2 | .906 | .011 | .885, .927 |
| Interaction | Inhibitory S1 | .791 | .016 | .759, .824 |
|  | Inhibitory S2 | .821 | .020 | .782, .861 |
|  | Prepotent S1 | .974 | .003 | .967, .981 |
|  | Prepotent S2 | .991 | .004 | .983, .999 |
| Reaction Time (ms) | | **Estimated marginal mean** | **Standard error** | **Confidence interval** |
| Trial type | Inhibitory | 1581 | 37 | 1508, 1653 |
|  | Prepotent | 1281 | 34 | 1213, 1349 |
| Session | 1 | 1647 | 45 | 1558, 1736 |
|  | 2 | 1215 | 29 | 1157, 1273 |
| Interaction | Inhibitory S1 | 1766 | 51 | 1665, 1867 |
|  | Inhibitory S2 | 1395 | 34 | 1328, 1463 |
|  | Prepotent S1 | 1528 | 47 | 1435, 1621 |
|  | Prepotent S2 | 1034 | 29 | 977, 1091 |
|  | | | | |

- - 1. **Test Re-Test Reliability**

To establish test re-test reliability, five confirmatory correlational analyses were conducted on data from the behavioural ECITT (Session 1) and the blocked ECITT (Session 2), results are reported in **Supplementary Table 14** below. Performance (as measured by the mean inhibitory accuracy variable, the accuracy inhibitory score and the median inhibitory reaction time variable) was consistent across sessions.

**Supplementary Table 14.** ECITT Test Re-Test Reliability.

|  | Pearson’s correlation coefficient | *p* | Confidence interval |
| --- | --- | --- | --- |
| Mean inhibitory accuracy | *r* (94) = .446 | <.001 | .270, .595 |
| Mean prepotent accuracy | *r* (94) = .164 | .054 | .044, .505 |
| Accuracy inhibitory | *r* (94) = .424 | <.001 | .266, .585 |
| Median inhibitory reaction time | *r* (94) = .464 | <.001 | .285, .639 |
| Median prepotent reaction time | *r* (94) = .060 | .278 | .001, .235 |
| *Note.* Statistically significant results are highlighted in bold. All results remained significant following the procedure for controlling the FDR (*N* = 5 comparisons). | | | |

- - 1. **Group Performance Differences**

Two 2 × 2 mixed ANOVAs were conducted to examine whether there were any significant performance differences between those participants who contributed valid fNIRS data and valid ECITT data (*N* = 61) and those who only contributed valid ECITT data (*N* = 39). Estimated marginal means are reported in **Supplementary Table 15**.

Accuracy: As hypothesised (H3a), results demonstrated a significant main effect of trial type, whereby participants were significantly more accurate on prepotent trials than on inhibitory trials: *F* (1, 98) = 81.484, *p* < .001, ηp^2^ = .454, Cohen’s *f* = 0.897. Again, as predicted, there was no significant main effect of sub-sample: *F* (1, 98) = .008, *p* = .927, ηp^2^ = .000, Cohen’s *f* = .000, and no significant trial type × sub-sample interaction: *F* (1, 98) = .259, *p* = .612, ηp^2^ = .003, Cohen’s *f* = .000.

Reaction Time: In line with predictions (H3b), results demonstrated a significant main effect of trial type, whereby participants responded significantly faster on correct prepotent trials than on correct inhibitory trials: *F* (1, 98) = 274.803, *p* < .001, ηp^2^ = .737, Cohen’s *f* = 1.655. There was also a significant main effect of sub-sample, whereby participants who contributed both valid ECITT and fNIRS data responded significantly faster than those who only contributed valid ECITT data: *F* (1, 98) = 5.203, *p* = .025, ηp^2^ = .050, Cohen’s *f* = 0.21. The trial type × sub-sample interaction was also significant, whereby participants who contributed valid fNIRS data were significantly faster on inhibitory trials than those who did not contribute valid fNIRS data: *F* (1, 98) = 8.694, *p* = .004, ηp^2^ = .081, Cohen’s *f* = 0.277.

**Supplementary Table 15.** Estimated Marginal Means for Mixed ANOVAs (Group Performance Differences).

| Accuracy | | Estimated marginal mean | Standard error | Confidence interval |
| --- | --- | --- | --- | --- |
| Trial type | Inhibitory | .811 | .020 | .770, .852 |
|  | Prepotent | .990 | .004 | .982, .998 |
| Sub-sample | Behavioural only | .899 | .017 | .865, .933 |
|  | fNIRS + Behavioural data | .901 | .014 | .874, .929 |
| Interaction | Behavioural only - Inhibitory | .815 | .032 | .752, .879 |
|  | Behavioural only - Prepotent | .984 | .006 | .971, .996 |
|  | fNIRS + Behavioural Data - Inhibitory | .807 | .026 | .756, .858 |
|  | fNIRS + Behavioural Data Prepotent | .996 | .005 | .986, 1.00 |
| Reaction time (ms) | | **Estimated marginal mean** | **Standard error** | **Confidence interval** |
| Trial type | Inhibitory | 1411 | 32 | 1347, 1475 |
|  | Prepotent | 1031 | 29 | 974, 1088 |
| Sub-sample | Behavioural only | 1286 | 44 | 1198, 1374 |
|  | fNIRS + Behavioural data | 1157 | 35 | 1086, 1227 |
| Interaction | Behavioural only - Inhibitory | 1510 | 51 | 1409, 1610 |
|  | Behavioural only - Prepotent | 1062 | 45 | 973, 1151 |
|  | fNIRS + Behavioural Data - Inhibitory | 1313 | 40 | 1233, 1393 |
|  | fNIRS + Behavioural Data Prepotent | 1000 | 36 | 929, 1072 |
|  | | | | |

## **Results of Non-Parametric Tests**

ECITT Condition Effects

H1 & H2: Results of non-parametric Wilcoxon signed rank tests supported the hypotheses that participants would be significantly more accurate on prepotent trials than on inhibitory trials (H1): *Z* = 6.993, *p* < .001, and that participants would respond significantly faster on correct prepotent trials than on correct inhibitory trials (H2): *Z* = -8.431, *p* <.001. Results aligned with the results of the parametric paired *t*-tests reported in the manuscript.

Behavioural Development from Infancy to Early Childhood

Accuracy: Results of non-parametric related-samples Wilcoxon Signed Rank tests revealed that there was a significant difference in accuracy (mean prepotent accuracy, mean inhibitory accuracy, accuracy inhibitory score) from 10-months to 3½ years (H6a), and from 16-months to 3½ years (H6b), whereby participants were significantly more accurate at 3½ years than at 10- or 16-months. Results are reported in **Supplementary Table 16** below and are in alignment with the parametric tests.

Reaction Time: Results of non-parametric related-samples Wilcoxon Signed Rank tests revealed that there was a significant difference in reaction time from 16-months to 3½ years (H6b), whereby participants responded significantly faster at 3½ years than at 16-months. Results are reported in **Supplementary Table 16** below and are in alignment with the parametric tests).

**Supplementary Table 16.** Non-Parametric Longitudinal Analyses at 3½ years (ECITT).

|  | 10-months and 3½ years | | 16-months and 3½ years | |
| --- | --- | --- | --- | --- |
|  | N | Statistic | N | Statistic |
| Mean inhibitory accuracy | 79 | Z = 4.996, p <.001 | 44 | Z = 3.206, p = .001 |
| Mean prepotent accuracy | 79 | Z = 6.085, p <.001 | 44 | Z = 4.694, p < .001 |
| Accuracy inhibitory | 78 | Z = 3.785, p <.001 | 44 | Z = 2.544, p = .011 |
| Median inhibitory reaction time | - | - | 42 | Z = -2.895, p = .004 |
| Median prepotent reaction time | - | - | 42 | Z = -5.133, p < .001 |
| *Note.* All results remained significant following the procedure for controlling the FDR (N = 8 comparisons). | | | | |

H7: Four separate Spearman’s correlational analyses were conducted to examine whether there is a significant association between performance on the blocked ECITT at 10-months and 3½ years (H7a) and at 16-months and 3½ years (H7b). Results are reported in **Supplementary Table 17** and are in alignment with the results of the parametric tests.

**Supplementary Table 17.** Performance on the ECITT at 3½ years: Longitudinal Associations.

|  | Statistic |
| --- | --- |
| Accuracy Inhibitory Score (10-months and 3½ years) | *r_s_* (76) = -.037, *p* = .375 |
| Accuracy Inhibitory Score (16-months and 3½ years) | *r_s_* (40) = -.222, *p* = .082 |
| Median Prepotent Reaction Time | *r_s_* (40) = .015, *p* = .463 |
| Median Inhibitory Reaction Time | *r_s_* (40) = .224, *p* = .086 |

*Additional Exploratory (Not Pre-Registered) Longitudinal Analyses*

It was pre-registered that two separate linear mixed models would be conducted to investigate the change in accuracy performance on the ECITT across assessment points (results are reported in the manuscript). Model 1 examined change from 10-months to 3½ years, and Model 2 examined change from 16-months to 3½ years. As predicted, the results indicated that there was a significant main effect of age in both models (such that participants were significantly more accurate at 3½ years than at 10- or 16-months of age) and that there was a significant main effect of trial type in both models (such that participants were significantly more accurate on prepotent trials than on inhibitory trials). There was also a significant interaction effect in both models, whereby there was a larger improvement in inhibitory accuracy than in prepotent accuracy between 10 months and 3½ years and between 16-months and 3½ years.

An additional exploratory (not pre-registered) 3 × 2 linear mixed model was conducted with the accuracy data from the ECITT as the dependent variable, and assessment point (10-months, 16-months and 3 ½ years) and trial type (prepotent, inhibitory) as the within-subject factors. Estimated marginal means are reported in **Supplementary Table 18** below. This was done to establish a full picture of accuracy development across infancy and early childhood. The results of the model suggest that there was a significant main effect of assessment point: *F* (2, 239.184) = 62.988, *p*< .001, Cohen’s *f* = 0.72. Results of Bonferroni-corrected post-hoc tests revealed that there was no significant difference between the estimated marginal mean for accuracy at 10-months and 16-months: mean difference = -.005, *SE* = .023, *df* = 181.518, *p* = 1.00, *CI* = -.050, .061. However, there was a significant improvement from 10-months to 3½ years: mean difference = .165, *SE* = .017, *df* = 251.907, *p*<.001, *CI* = .125, .206, and from 16-months to 3 ½ years: mean difference = .170, *SE* = .021, *df* = 143.137, *p* <.001, *CI* = .119, .222.

A significant main effect of trial type was also found *F* (1, 243.369) = 351.845, *p* <.001, Cohen’s *f* = 1.20, such that accuracy was significantly higher on prepotent trials than on inhibitory trials, as would be expected. Finally, there was a significant assessment point × trial type interaction: *F* (2, 239.184) = 22.112, *p* <.001, Cohen’s *f* = 0.42. This suggests that there was a larger accuracy improvement on inhibitory trials than on prepotent trials across assessment points.

**Supplementary Table 18.** Estimated Marginal Means: Accuracy Improvement from Infancy to Early Childhood.

|  | | Estimated marginal mean | Standard error | Degrees of Freedom | Confidence interval |
| --- | --- | --- | --- | --- | --- |
| Trial Type | Inhibitory | .632 | .016 | 213.409 | .600, .664 |
|  | Prepotent | .946 | .004 | 199.159 | .937, .954 |
| Age | 10-months | .735 | .013 | 148.395 | .709, .762 |
|  | 16-months | .730 | .019 | 91.636 | .693, .767 |
|  | 3½ years | .901 | .010 | 106.763 | .880, .921 |
| Interaction | Inhibitory 10-months | .549 | .026 | 127 | .497, .600 |
|  | Prepotent 10-months | .922 | .008 | 127 | .907, .937 |
|  | Inhibitory 16-months | .536 | .036 | 80 | .465, .608 |
|  | Prepotent 16-months | .924 | .010 | 80 | .905, .943 |
|  | Inhibitory 3½ years | .810 | .020 | 99 | .771, .999 |
|  | Prepotent 3½ years | .991 | .004 | 99 | .983, .999 |

Between-Session Consistency and Test Re-Test Reliability

H4: Results of non-parametric related-samples Wilcoxon Signed Rank tests revealed that there was a significant difference in prepotent accuracy between sessions, whereby participants were significantly more accurate in Session 2 (*M* = .991, *SD* = .039) than in Session 1 (*M* = .973, *SD* = .036): *Z* = 5.598, *p* < .001. However, there was no significant difference in inhibitory accuracy between sessions: *Z* = 1.845, *p* = .065. There was also no significant difference in accuracy inhibitory score between sessions: *Z* = 1.148, *p* = .251.

For the reaction time variables, results of non-parametric related-samples Wilcoxon Signed Rank tests revealed that there was a significant difference in prepotent reaction time between sessions, whereby participants were significantly faster in Session 2 (*M* = 1024 ms, *SD* = 280 ms) than in Session 1 (*M* = 1496 ms, *SD* = 437 ms): *Z* = -8.259, *p* < .001. Similarly, there was a significant difference in inhibitory reaction time between sessions, whereby participants were significantly faster in Session 2 (*M* = 1378 ms, *SD* = 328 ms) than in Session 1 (*M* = 1757 ms, *SD* = 489 ms): *Z* = -7.342, *p* < .001.

Overall, these results suggest that participants responded significantly faster on both trial types in Session 2 than in Session 1 and that participants were significantly more accurate on prepotent trials (but not inhibitory trials) in Session 2 than in Session 1.

To establish test re-test reliability, five confirmatory correlational analyses were conducted on data from the behavioural ECITT (Session 1) and the blocked ECITT (Session 2), results are reported in **Supplementary Table 19** below and support the hypotheses (H5). However, there is some convergence from the results of parametric correlation analyses reported in Supplementary Materials 5.2.

**Supplementary Table 19.** ECITT Test Re-test Reliability at 3½ years.

|  | Spearman’s correlation coefficient | *p* | Confidence interval |
| --- | --- | --- | --- |
| Mean inhibitory accuracy | *r* (94) = .452 | **<.001** | .254, .623 |
| Mean prepotent accuracy | *r* (94) = .217 | **.017** | -.012, .413 |
| Accuracy inhibitory | *r* (94) = .428 | **<.001** | .250, .592 |
| Median inhibitory reaction time | *r* (94) = .552 | **<.001** | .378, .689 |
| Median prepotent reaction time | *r* (94) = .174 | **.042** | -.004, .345 |
| *Note.* Statistically significant results are highlighted in bold. All results remained significant following the procedure for controlling the FDR (*N* = 5 comparisons). | | | |

ECITT Group Performance Differences

H3a: Results of non-parametric Mann-Whitney U tests revealed that there was no significant difference in inhibitory accuracy between groups (those with fNIRS data: *N* = 61 and those without fNIRS data: *N* = 39): *Z* = .196, *p* = .845, however does suggest that participants who contributed valid fNIRS data were significantly more accurate on prepotent trials (mean rank = 53.61) than those who did not contribute valid fNIRS data (mean rank = 45.64): *Z* = 1.999, *p* = .046. This does not align with the results of parametric tests.

H3b: Results of non-parametric Mann-Whitney U tests revealed that there was no significant difference in median prepotent reaction time between groups: *Z* = -.671, *p* = .502, however there was evidence to suggest that participants who contributed valid fNIRS data were significantly faster at responding on inhibitory trials (mean rank = 44.93) than those without valid fNIRS data (mean rank = 59.21): *Z* = -2.399, *p* = .016. This aligns with parametric test results.

Individual Differences Analyses

In line with the [pre-registration](https://www.dropbox.com/scl/fo/1o829pmwigdfhnwqddamr/h?rlkey=dqrttfk8xcauf2ahh2ixx4opo&dl=0), exploratory correlational analyses were conducted to investigate whether individual differences in neural activation in specific channels (showing significant block type effects across the identified time-bins) were associated with individual performance differences in the accuracy inhibitory score. Results of Spearman’s correlational analyses (**Supplementary Table 20**) revealed that there were no significant associations between performance on the ECITT (accuracy inhibitory score) and the haemoglobin difference variables. These results were in convergence with the results of the parametric Pearson’s correlational analyses.

**Supplementary Table 20.** Individual Differences Analyses at 3½ years.

| Location | Hemisphere | Channel | Signal | Time Bins | Statistic |
| --- | --- | --- | --- | --- | --- |
| Inferior parietal | Right | 7 | HHb | 5 | *r_s_* (35) = -.088, *p* = .612, *CI* = -.452, .266 |
|  |  | **8*** | HHb | 4 – 5 | *r_s_* (32) = -.198, *p* = .268, *CI* = -.509, .153 |
| IFG | Right | **31*** | HbO_2_ | 1 – 2 | *r_s_* (51) = .006, *p* = .968, *CI* = -.291, .310 |
|  |  |  | HHb | 3 – 5 | *r_s_* (51) = .067, *p* = .636, *CI* = -.206, .344 |
| DLPFC | Left | 18 | HbO_2_ | 3 – 4 | *r_s_* (49) = -.125, *p* = .388, *CI* = -.411, .149 |
|  |  | 43 | HHb | 1 – 3 | *r_s_* (40) = -.054, *p* = .738, *CI* = -.359, .246 |
|  | Right | 45 | HbO_2_ | 1 – 2 | *r_s_* (46) = -.160, *p* = .283, *CI* = -.453, .149 |
| OFC | Left | **29*** | HHb | 2 | *r_s_* (56) = -.146, *p* = .279, *CI* = -.373, .128 |
| *Note.* IFG = inferior frontal gyrus, DLPFC = dorsolateral prefrontal cortex, OFC = orbital frontal cortex. | | | | | |

## **fNIRS Group-Level Results**

### **Main Effect of Time**

**Supplementary Table 21** and **Supplementary Table 22** display the results of repeated measures ANOVAs for channels showing a significant main effect of time (a significant increase in HbO_2_ / decrease in HHb from baseline).

**Supplementary Table 21.** Main Effect of Time (HbO2) in the 3½ year Dataset.

|  |  | Left hemisphere | |  | Right hemisphere | |
| --- | --- | --- | --- | --- | --- | --- |
| IPS | Channel | *F* | *p* | Channel | *F* | *p* |
|  | 1 | 8.228 | **<.001^*^** | 7 | 7.273 | **.001^*^** |
|  | 4 | 1.553 | .217 | 8 | 2.497 | .085 |
| Prefrontal Cortex | 13 | 3.797 | **.028^*^** | 20 | .990 | .375 |
|  | 14 | 3.693 | **.037** | 21 | 3.305 | **.037** |
|  | 16 | 8.942 | **<.001^*^** | 23 | 6.924 | **.002^*^** |
|  | 17 | 27.417 | **<.001^*^** | 25 | 26.162 | **<.001^*^** |
|  | 18 | 14.582 | **<.001^*^** | 26 | 36.350 | **<.001^*^** |
|  | 19 | 26.901 | **<.001^*^** | 31 | 16.935 | **<.001^*^** |
|  | 27 | 13.568 | **<.001^*^** | 32 | 45.233 | **<.001^*^** |
|  | 28 | 18.153 | **<.001^*^** | 33 | 30.806 | **<.001^*^** |
|  | 29 | 9.767 | **<.001^*^** | 37 | 23.455 | **<.001^*^** |
|  | 35 | 3.325 | **.040** | 38 | 44.366 | **<.001^*^** |
|  | 36 | 3.129 | .053 | 41 | 21.858 | **<.001^*^** |
|  | 39 | .679 | .509 | 42 | 14.578 | **<.001^*^** |
|  | 40 | .678 | .498 | 45 | 14.407 | **<.001^*^** |
|  | 43 | .185 | .828 | 46 | 4.285 | **.023^*^** |
|  | 44 | .784 | .463 |  |  |  |
| *Note.* Statistically significant results are highlighted in bold. * = remained significant following the procedure for controlling the FDR (*N* = 16 comparisons; 8 confirmatory channels × 2 chromophores) (*N* = 50 comparisons, 25 exploratory channels × 2 chromophores). | | | | | | |

**Supplementary Table 22.** Main Effect of Time (HHb) in the 3½ year Dataset.

|  |  | Left hemisphere | |  | Right hemisphere | |
| --- | --- | --- | --- | --- | --- | --- |
| IPS | Channel | *F* | *p* | Channel | *F* | *p* |
|  | 1 | .791 | .477 | 7 | 1.099 | .342 |
|  | 4 | .961 | .381 | 8 | 1.533 | .220 |
| Prefrontal Cortex | 13 | 5.421 | **.006^*^** | 20 | .542 | .614 |
|  | 14 | 5.323 | **.007^*^** | 21 | 2.402 | .099 |
|  | 16 | 2.597 | .089 | 23 | .878 | .422 |
|  | 17 | 4.795 | **.011^*^** | 25 | 7.359 | **.001^*^** |
|  | 18 | 1.743 | .171 | 26 | 11.470 | **<.001^*^** |
|  | 19 | 6.863 | **.001^*^** | 31 | 4.659 | **.010^*^** |
|  | 27 | 4.284 | **.021^*^** | 32 | 19.882 | **<.001^*^** |
|  | 28 | 22.733 | **<.001^*^** | 33 | 13.569 | **<.001^*^** |
|  | 29 | 9.059 | **<.001^*^** | 37 | 3.726 | **.029^*^** |
|  | 35 | 2.866 | .068 | 38 | 6.574 | **.003^*^** |
|  | 36 | 4.840 | **.013^*^** | 41 | 5.533 | **.011^*^** |
|  | 39 | 4.266 | **.024^*^** | 42 | 10.605 | **<.001^*^** |
|  | 40 | 5.848 | **.003^*^** | 45 | 16.935 | **<.001^*^** |
|  | 43 | 7.245 | **<.001^*^** | 46 | 12.303 | **<.001^*^** |
|  | 44 | 7.762 | **<.001^*^** |  |  |  |
| *Note*. Statistically significant results are highlighted in bold. * = remained significant following the procedure for controlling the FDR (*N* = 16 comparisons; 8 confirmatory channels × 2 chromophores) (*N* = 50 comparisons, 25 exploratory channels × 2 chromophores). | | | | | | |

### **Excluded Channels**

It was found during pre-processing of the fNIRS data that there were 10 channels for which less than 50% of participants contributed data (bilateral parietal: Channels 2, 3, 5, 9, 10, 11, 12, and bilateral middle frontal gyrus: 15, 22, and 24). These channels were excluded from the analysis. **Supplementary Figure 5** highlights where these channels were located on the fNIRS probe. These channels likely had poor optode coupling due to the position on the curve of the head and/or because of hair in these areas. Additionally, the two short separation channels (Channels 30 and 34) were excluded from the analysis because they were showing significant block type effects and so were not functioning as short channels.

**Supplementary Figure 5.** Excluded Channels Displayed on the fNIRS Probe.

| 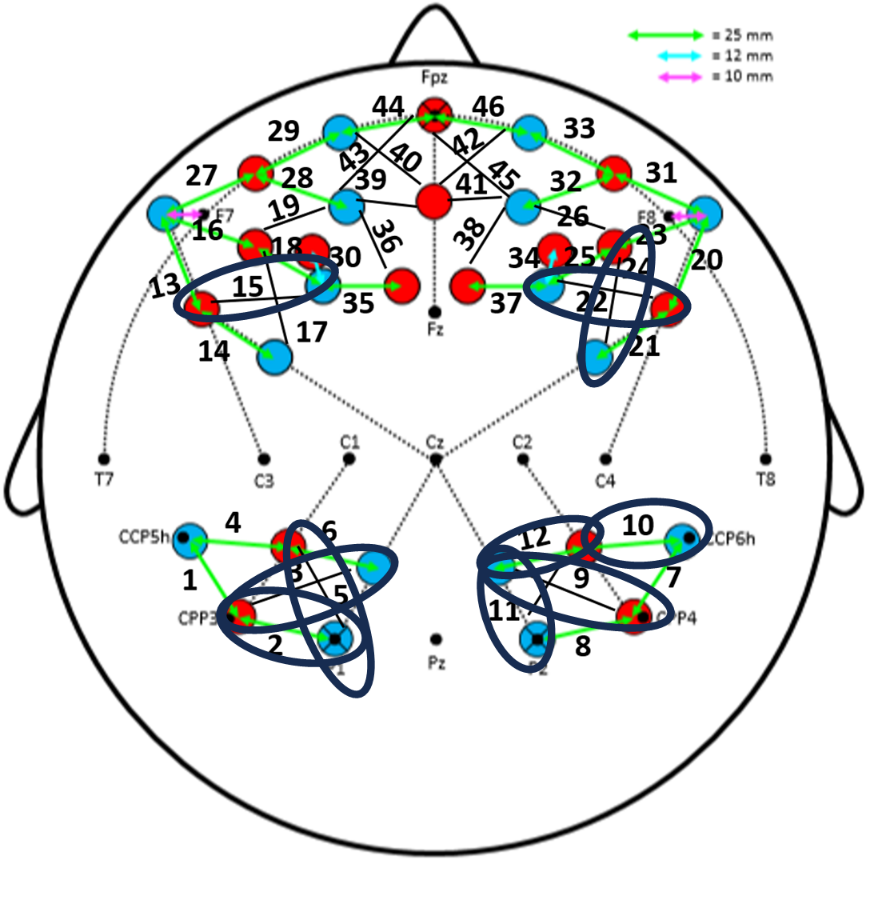 |
| --- |

### **Time Course of the Significant Block-Type Effect**

To examine the time course of the significant effects, paired t-tests were conducted on the seven channels identified in the primary analyses as showing significant block-type effects; results are reported in **Supplementary Table 23**. It is worth noting that for Channel 7 (right inferior parietal) and Channel 29 (left OFC) there were no significant HHb concentration differences between block types within any of the five time bins. This is because the paired t-tests were conducted on an exploratory basis using the two-tailed p-values. As such, no further analyses will be conducted with data from these channels.

**Supplementary Table 23.** Time Course of the Significant HbO_2_ or HHb Block Type Effects.

| Location | Channel | Signal | 0 – 4s | 4 – 8s | 8 – 12s | 12 – 16s | 16 – 20s |
| --- | --- | --- | --- | --- | --- | --- | --- |
| Right inferior parietal | 7 | HHb | *t* (35) = -.308,  *p* = .760,  *d* = .051 | *t* (35) = .207,  *p* = .837,  *d* = .034 | *t* (35) = 1.076,  *p* = .289,  *d* = .179 | *t* (35) = 1.682,  *p* = .102,  *d* = .280 | *t* (35) = 1.946,  *p* = .060,  *d* = .324 |
|  | 8* | HHb | *t* (32) = 1.035,  *p* = .308,  *d* = .180 | *t* (32) = 1.005,  *p* = .322,  *d* = .175 | *t* (32) = 1.347,  *p* = .187,  *d* = .235 | *t* (32) = 2.089,  *p* = .045,  *d* = .364 | *t* (32) = 2.245,  *p* = .032,  *d* = .391 |
| Right IFG | 31* | HbO_2_ | t (51) = 2.826,  p = .007_#_,  d = .392 | t (51) = 2.403,  p = .020_#_,  d = .333 | t (51) = 1.823,  p = .074,  d = .253 | t (51) = 1.478,  p = .146,  d = .205 | t (51) = 1.594,  p = .117,  d = .221 |
|  |  | HHb | *t* (51) = 1.594,  *p* = .117,  *d* = .221 | *t* (51) = 1.656,  *p* = .104,  *d* = 230 | *t* (51) = 2.214,  *p* = .031,  *d* = .307 | *t* (51) = 2.152,  *p* = .036,  *d* = .298 | *t* (51) = 2.082,  *p* = .042,  *d* = .289 |
| Left DLPFC | 18 | HbO_2_ | *t* (49) = .749,  *p* = .458,  *d* = .106 | *t* (49) = 2.005,  *p* = .051,  *d* = .284 | *t* (49) = 2.460,  *p* = .017,  *d* = .348 | *t* (49) = 2.365,  *p* = .022,  *d* = .334 | *t* (49) = 1.889,  *p* = .065,  *d* = .267 |
|  | 43 | HHb | *t* (40) = -2.488,  *p* = .017_#_,  *d* = .389 | *t* (40) = -2.499,  *p* = .017_#_,  *d* = .390 | *t* (40) = -2.788,  *p* = .008_#_,  *d* = .435 | *t* (40) = -2.002,  *p* = .052,  *d* = .313 | *t* (40) = -1.384,  *p* = .174,  *d* = .216 |
| Right DLPFC | 45 | HbO_2_ | *t* (46) = 3.082,  *p* = .003_#_,  *d* = .450 | *t* (46) = 2.206,  *p* = .032,  *d* = .322 | *t* (46) = 1.978,  *p* = .054,  *d* = .289 | *t* (46) = 1.893,  *p* = .065,  *d* = .276 | *t* (46) = 1.337,  *p* = .188,  *d* = .195 |
| Left OFC | 29* | HHb | *t* (56) = -.399,  *p* = .691,  *d* = .053 | *t* (56) = -1.752,  *p* = .085,  *d* = .232 | *t* (56) = -1.614,  *p* = .112,  *d* = .214 | *t* (56) = -.760,  *p* = .450,  *d* = .101 | *t* (56) = .113,  *p* = .910,  *d* = .015 |
| *Note.* * = Channels were pre-registered in the confirmatory analyses (and also showed significant effects at 16-months). Statistically significant results are highlighted in bold. ^#^ = significant after correcting the FDR (5 comparisons). The significant effects in Channel 31 (HbO_2_), Channel 18, Channel 43, Channel 45 and Channel 29 are in the opposite direction than expected. IFG = inferior frontal gyrus, DLPFC = dorsolateral prefrontal cortex, OFC = orbital frontal cortex. | | | | | | | |
|  |  |  |  |  |  |  |  |

### **Description of the Significant Block Type Effects**

As seen in **Table 4 of the manuscript**, a significant time × block type interaction was found in the HHb signal for Channel 7 and Channel 8 (right inferior parietal), suggesting that there was a significantly greater decrease in HHb concentration in experimental blocks compared to in control blocks, and that the HHb difference between conditions was dependent on the time bin. A significant main effect of block type was observed in the HHb signal for Channel 31 (right IFG orbital), such that there was a significantly greater HHb decrease in experimental blocks than in control blocks. These effects suggest that there is a significantly greater HHb decrease in the right inferior parietal cortex (Channels 7 and 8) and the right IFG (Channel 31) when inhibition is required.

Additionally, a significant HbO_2_ block type effect was observed for Channel 18 (left DLPFC), Channel 31 (right IFG orbital) and Channel 45 (right DLPFC). However, the effects were in the opposite direction than predicted, as HbO_2_ concentration showed a significantly greater increase in control blocks than in experimental blocks. Similarly, a significant HHb block type effect was observed for Channel 43 (left DLPFC), and a significant HHb time × block type interaction effect was observed for Channel 29 (left OFC), but again these effects were not in the predicted direction as there was a significantly greater HHb decrease in control blocks compared to experimental blocks.

As can be seen in **Figure 5 of the manuscript,** Channel 7 (right inferior parietal) and Channel 29 (left OFC) show an inverted HRF response. In Channel 7, there is an overall decrease in both HbO_2_ and HHb from baseline, whereby the HbO_2_ concentration (in both block types) is lower than the HHb concentration. After approximately 10 s of the block time course, the response appears to correct such that there is an increase in HbO_2_ (in both block types), however, the HHb signal appears to remain fairly steady until around 16 s of the block time course where the HHb in control blocks increases and there is a significant (one-tailed) HHb difference between block types (from 16 – 20 s). Overall, the effects found in Channel 7 suggest that there was a larger HHb decrease in the experimental compared to control blocks, as expected. In Channel 29, the inversion occurs early in the block time course until around 12s where the HbO_2_ signal (in both block types) begins to increase, and from around 8s the HHb signal (in both block types) begins to decrease. The significant interaction found in Channel 29 occurs from 4 – 8s of the block time course (one-tailed). Overall, the effects found in Channel 29 suggest that there was a larger increase in HHb concentration in experimental blocks than in control blocks. Not only is the HHb signal increasing from baseline when the significant effect is observed (when it is expected to decrease from baseline), but it is also the case that there was a larger increase in experimental compared to control blocks (where it would be expected that there was a *lower* HHb concentration in experimental blocks).

### **Individual Differences (Brain-Behaviour) Analyses**

Exploratory correlational analyses were conducted to investigate whether individual differences in neural activation (in channels showing significant block type effects across the identified time bins) were associated with individual performance differences in the accuracy inhibitory score. Results of Pearson’s correlational analyses (**Supplementary Table 24**) revealed that there were no significant associations between performance on the ECITT (accuracy inhibitory score) and the haemoglobin difference variables.

**Supplementary Table 24.** Individual Differences Analyses.

| Location | Hemisphere | Channel | Signal | Time Bins | Statistic |
| --- | --- | --- | --- | --- | --- |
| Inferior parietal | Right | 8* | HHb | 4 – 5 | *r* (32) = -.143, *p* = .427, *CI* = -.439, .168 |
| IFG | Right | 31* | HbO_2_ | 1 – 2 | *r* (51) = -.043, *p* = .760,  *CI* = -.334, .266 |
|  |  |  | HHb | 3 – 5 | *r* (51) = .090, *p* = .527,  *CI* = -.196, .382 |
| DLPFC | Left | 18 | HbO_2_ | 3 – 4 | *r* (49) = -.007, *p* = .962,  *CI* = -.341, .359 |
|  |  | 43 | HHb | 1 – 3 | *r* (40) = -.087, *p* = .590,  *CI* = -.378, .241 |
|  | Right | 45 | HbO_2_ | 1 – 2 | *r* (46) = -.162, *p* = .276,  *CI* = -.429, .140 |
| *Note.* * = Channels were pre-registered in the confirmatory analyses (and also showed significant effects at 16-months). IFG = inferior frontal gyrus, DLPFC = dorsolateral prefrontal cortex, OFC = orbital frontal cortex. | | | | | |

### **Exploratory Longitudinal fNIRS Analyses**

Since no channels showed significant block type effects at both 10-months and 3½ years, it was decided that the analyses examining change from 10-months to 3½ years would not be conducted (deviating from the pre-registered analysis plan). However, we did conduct exploratory (pre-registered) longitudinal analyses to examine how brain activation in channels found to show significant block type effects changes from 16-months to 3½ years.

- - 1. ***Channels Showing Significant Block Type Effects at 3½ years***

Separate linear mixed models (restricted maximum likelihood estimation) were conducted on the four channels showing a significant block type effect at 3½ years (dependent variable), with assessment point (16-months, 3½ years) as a fixed factor and participants as a random factor. A total of five models were conducted as Channel 31 showed significant block type effects in both HbO2 and HHb signals. Note that data from Channel 7 (right inferior parietal) and Channel 29 (left OFC) were not included in these analyses. The models were conducted to investigate the change in activation from the 16-month assessment point to the 3½ year assessment point. Results indicated that the HbO_2_ difference in Channel 18 (left DLPFC) significantly increased from 16-months to 3½ years, such that there was a larger HbO_2_ difference at 3½ years compared to 16-months (**Supplementary Table 25,** estimated marginal means are reported in **Supplementary Table 26**). In other words, whilst there was no significant difference in HbO_2_ concentration between block types at 16-months, there was a significant difference at 3½ years, such that the HbO_2_ concentration was significantly higher in control blocks than in experimental blocks (note that this is in the *opposite* direction than expected, hence the negative estimated marginal mean at 3½ years). As such, the change appears negative (**Supplementary Figure 6**) due to the way that the HbO_2_ difference measure is calculated (HbO_2_ concentration in experimental blocks *minus* HbO_2_ concentration in control blocks), but reflects a larger difference between block types at 3½ years (in the opposite direction than expected) compared to no significant difference between block types at 16-months.

- - 1. ***Channels Showing Significant Block Type Effects at 16-months***

Six additional linear mixed models were separately conducted to investigate the change in activation across assessment points in channels showing significant block-type effects at 16-months, but not at 3½ years. Results indicated that the HHb difference in Channel 28 significantly decreased from 16-months to 3½ years (**Supplementary Figure 7**), such that there was a significantly larger HHb difference at 16-months (where there was a significantly greater HHb decrease in experimental blocks than in control blocks) compared to at 3½ years (where no significant block type effect was found) (see also **Supplementary Table 25**, estimated marginal means are reported in **Supplementary Table 27**).

**Supplementary Table 25.** Exploratory Longitudinal Brain Changes in Channels with a Significant Block Type Effect.

| Channels with a Significant Block Type Effect at 3½ years | | | | | | |
| --- | --- | --- | --- | --- | --- | --- |
| Location | Hemisphere | Channel | Signal | Statistic | *P* | Effect Size (Cohen’s f) |
| Inferior parietal | Right | 8* | HHb | *F* (1, 53.291) = .180 | .673 | .000 |
| IFG | Right | 31* | HbO_2_ | *F* (1, 86.571) = 1.930 | .168 | .102 |
|  |  |  | HHb | *F* (1, 85.014) = .013 | .909 | .000 |
| DLPFC | Left | 18 | HbO_2_ | *F* (1, 71.844) = 5.235 | .025 | .239 |
|  |  | 43 | HHb | *F* (1, 69.984) = 3.466 | .067 | .185 |
|  | Right | 45 | HbO_2_ | *F* (1, 69.881) = 1.704 | .196 | .099 |
| Channels with a Significant Block Type Effect at 16-months | | | | | | |
| Location | Hemisphere | Channel | Signal | Statistic | *P* | Effect Size (Cohen’s f) |
| Superior parietal | Left | 6 | HHb | *F* (1, 32.262) = .162 | .690 | .000 |
| IFG | Right | 20 | HHb | *F* (1, 65.680) = .828 | .366 | .000 |
| IFG | Right | 23 | HHb | *F* (1, 80.699) = .674 | .414 | .000 |
| DLPFC | Left | 28 | HHb | *F* (1, 90.869) = 5.097 | .026 | .210 |
| DLPFC | Right | 26 | HbO_2_ | *F* (1, 74.297) = 1.291 | .260 | .062 |
| OFC | Right | 33 | HHb | *F* (1, 96.949) = 2.437 | .122 | .121 |
| *Note.* * = Channels also showed significant effects in the 16-month study (Fiske et al., 2024). The statistically significant results are highlighted in bold, although did not survive the procedure for controlling the FDR (Benjamini & Hochberg, 1995). IFG = inferior frontal gyrus, DLPFC = dorsolateral prefrontal cortex. | | | | | | |

**Supplementary Table 26.** Exploratory Longitudinal Brain Changes from 16-months; Estimated Marginal Means (channels with a significant block type effect at 3½ years).

| Location | Channel | Assessment Point | Estimated marginal mean | Standard error | df | Confidence interval |
| --- | --- | --- | --- | --- | --- | --- |
| Right inferior parietal | 8 (HHb) | 16-months | 1.221 | .564 | 35 | .075, 2.367 |
|  |  | 3½ years | 1.683 | .931 | 32 | -.213, 3.579 |
| Right IFG | 31 (HbO_2_) | 16-months | -.621 | .929 | 42 | -2.496, 1.254 |
|  |  | 3½ years | -2.917 | 1.367 | 51 | -5.661, -.173 |
|  | 31  (HHb) | 16-months | 1.581 | .419 | 42 | .736, 2.426 |
|  |  | 3½ years | 1.493 | .641 | 51 | .206, 2.780 |
| Left DLPFC | 18 (HbO_2_) | 16-months | .069 | .967 | 41 | -1.888, 2.027 |
|  |  | 3½ years | -4.826 | 1.908 | 49 | -8.660, -.993 |
|  | 43  (HHb) | 16-months | .282 | .655 | 35 | -1.048, 1.613 |
|  |  | 3½ years | -1.291 | .533 | 40 | -2.368, -.213 |
| Right DLPFC | 45 (HbO_2_) | 16-months | -.147 | .987 | 37 | -2.147, 1.854 |
|  |  | 3½ years | -1.731 | 707 | 46 | -3.154, -.309 |
| *Note.* Estimated marginal mean refers to the mean haemoglobin concentration difference between experimental and control blocks in each channel across the block time course. IFG = inferior frontal gyrus, DLPFC = dorsolateral prefrontal cortex. | | | | | | |

**Supplementary Table 27.** Exploratory Longitudinal Brain Changes; Estimated Marginal Means (channels with a significant block type effect at 16-months).

| Location | Channel | Assessment Point | Estimated marginal mean | Standard error | df | Confidence interval |
| --- | --- | --- | --- | --- | --- | --- |
| L superior parietal gyrus (HHb) | 6 | 16-months | 1.127 | .495 | 38 | .125, 2.129 |
|  |  | 3½ years | .537 | 1.112 | 23 | -1.663, 2.936 |
| R IFG (HHb) | 20 | 16-months | 1.046 | .480 | 41 | .076, 2.016 |
|  |  | 3½ years | -.034 | 1.086 | 48 | -2.217, 2.149 |
| R IFG (HHb) | 23 | 16-months | .970 | .474 | 42 | .013, 1.927 |
|  |  | 3½ years | .238 | .754 | 49 | -1.277, 1.754 |
| L DLPFC (HHb) | 28 | 16-months | 1.268 | .445 | 41 | .369, 2.166 |
|  |  | 3½ years | -.198 | .473 | 50 | -1.149, .752 |
| R DLPFC  (HbO_2_) | 26 | 16-months | 1.136 | 1.132 | 36 | -1.160, 3.432 |
|  |  | 3½ years | -.903 | 1.392 | 40 | -3.716, 1.911 |
| R OFC (HHb) | 33 | 16-months | 1.104 | .533 | 42 | .028, 2.180 |
|  |  | 3½ years | -.078 | .537 | 57 | -1.154, .998 |
| *Note.* Estimated marginal mean refers to the mean haemoglobin concentration difference between experimental and control blocks in each channel across the block time course. L = left, R = Right. IFG = inferior frontal gyrus, DLPFC = dorsolateral prefrontal cortex, OFC = orbital frontal cortex. | | | | | | |

**Supplementary Figure 6.** Exploratory Longitudinal Brain Changes (Channels Showing Significant Change from 16-months to 3½ years).

| 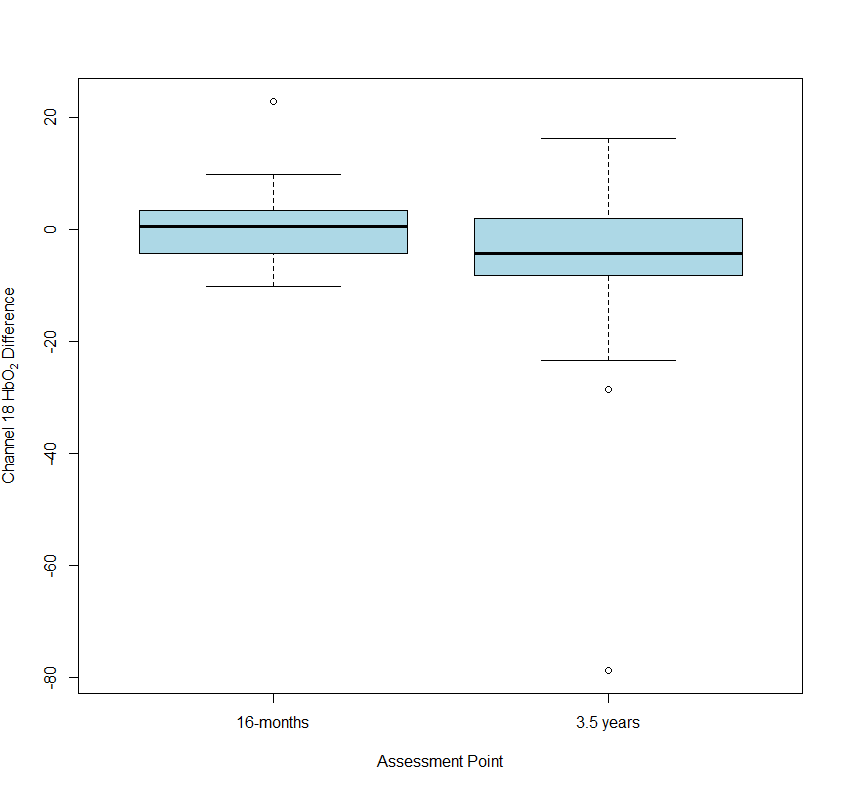 |
| --- |
| *Note.* This figure illustrates the change in activation from the 16-month assessment point to the 3½ year (42-month) assessment point in Channel 18 (HbO_2;_ left DLPFC), which showed a significant effect of assessment point (i.e., a significant change over time). Note that Channel 18 showed a significant block type effect at 3½ years (in the opposite direction to what was expected), but not at 16-months. The variable on the y-axis refers to the HbO_2_ difference score (calculated as HbO_2_ in experimental blocks minus HbO_2_ in control blocks) across the block time course. |

**Supplementary Figure 7.** Exploratory Longitudinal Brain Changes (Significant Block Type Effects at 16-months but not at 3½ years).

| 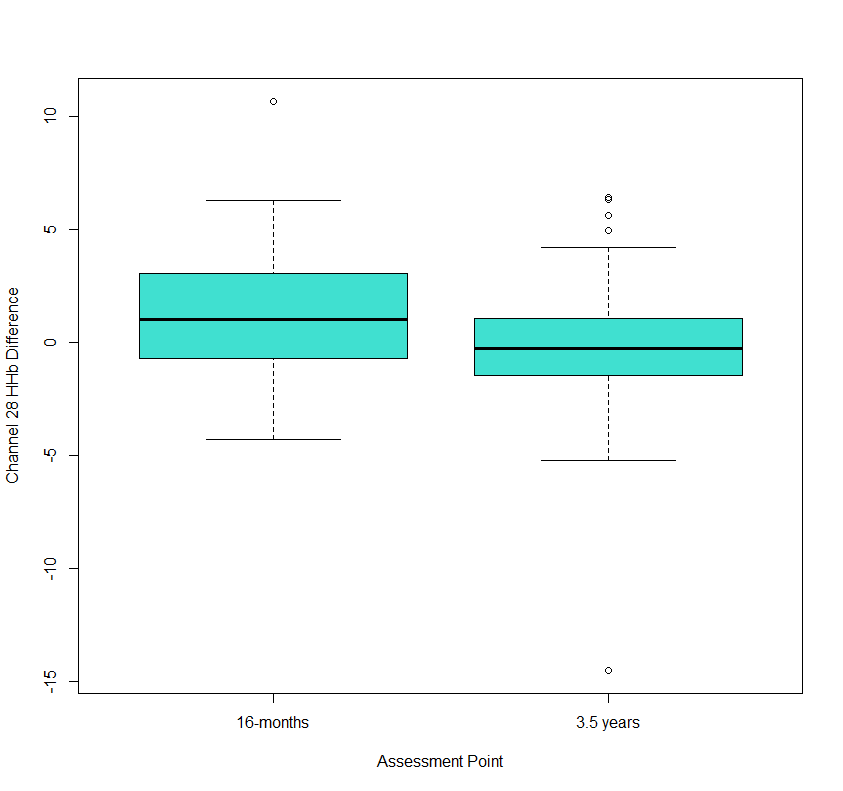 |
| --- |
| *Note.* This figure illustrates the decrease in activation from the 16-month assessment point to the 3½ year (42-month) assessment point in Channel 28 (HHb; left DLPFC), which showed a significant effect of assessment point (i.e., a significant change over time). Note that Channel 28 showed a significant block type effect at 16-months, but not at 3½ years. The variable on the y-axis refers to the HHb difference score (calculated as HHb in control blocks minus HHb in experimental blocks) across the block time course. |

## **References**

Aasted, C. M., Yücel, M. A., Cooper, R. J., Dubb, J., Tsuzuki, D., Becerra, L., Petkov, M. P., Borsook, D., Dan, I., & Boas, D. A. (2015). Anatomical guidance for functional near-infrared spectroscopy: AtlasViewer tutorial. Neurophotonics, 2(2), 020801. https://doi.org/10.1117/1.nph.2.2.020801

Benjamini, Y., & Hochberg, Y. (1995). Controlling the False Discovery Rate : A Practical and Powerful Approach to Multiple Testing. Journal of the Royal Statistical Society, 57(1), 289–300. https://doi.org/10.1111/j.2517-6161.1995.tb02031.x

Boas, D. A., & Dale, A. M. (2005). Simulation study of magnetic resonance imaging–guided cortically constrained diffuse optical tomography of human brain function. Applied Optics, 44(10), 1957–1968. https://doi.org/10.1364/AO.44.001957

Boas, D. A., Dale, A. M., & Franceschini, M. A. (2004). Diffuse optical imaging of brain activation: Approaches to optimizing image sensitivity, resolution, and accuracy. NeuroImage, 23(SUPPL. 1), S275–S288. https://doi.org/10.1016/j.neuroimage.2004.07.011

Brigadoi, S., Galderisi, A., Pieropan, E., Cooper, R. J., Cutini, S., Baraldi, E., Cobelli, C., Dell’Acqua, R., Sparacino, G., & Trevisanuto, D. (2019). Mapping hemodynamic changes during hypoglycemia in the very preterm neonatal brain: Preliminary results. Optics InfoBase Conference Papers, Part F142-ECBO 2019, 11074_13. https://doi.org/10.1117/12.2526974

Collins-Jones, L. H., Arichi, T., Poppe, T., Billing, A., Xiao, J., Fabrizi, L., Brigadoi, S., Hebden, J. C., Elwell, C. E., & Cooper, R. J. (2021). Construction and validation of a database of head models for functional imaging of the neonatal brain. Human Brain Mapping, 42(3), 567–586. https://doi.org/10.1002/hbm.25242

Fang, Q., & Boas, D. A. (2009). Tetrahedral mesh generation from volumetric binary and grayscale images. Proceedings - 2009 IEEE International Symposium on Biomedical Imaging: From Nano to Macro, ISBI 2009, 1142–1145. https://doi.org/10.1109/ISBI.2009.5193259

Fiske, A., Collins-Jones, L., de Klerk, C., Lui, K. Y. K., Hendry, A., Greenhalgh, I., Hall, A., Dvergsdal, H., Scerif, G., & Holmboe, K. (2024). The neural correlates of response inhibition across the transition from infancy to toddlerhood: An fNIRS study. Imaging Neuroscience, 2, 1–21. https://doi.org/10.1162/IMAG_A_00206

Fiske, A., de Klerk, C., Lui, K. Y. K., Collins-Jones, L., Hendry, A., Greenhalgh, I., Hall, A., Scerif, G., Dvergsdal, H., & Holmboe, K. (2022). The neural correlates of inhibitory control in 10-month-old infants: A functional near-infrared spectroscopy study. NeuroImage, 257. https://doi.org/10.1016/J.NEUROIMAGE.2022.119241

Frijia, E. M., Billing, A., Lloyd-Fox, S., Vidal Rosas, E., Collins-Jones, L., Crespo-Llado, M. M., Amadó, M. P., Austin, T., Edwards, A., Dunne, L., Smith, G., Nixon-Hill, R., Powell, S., Everdell, N. L., & Cooper, R. J. (2021). Functional imaging of the developing brain with wearable high-density diffuse optical tomography: A new benchmark for infant neuroimaging outside the scanner environment. NeuroImage, 225(October). https://doi.org/10.1016/j.neuroimage.2020.117490

Hendry, A., Greenhalgh, I., Bailey, R., Fiske, A., Dvergsdal, H., & Holmboe, K. (2021). Development of directed global inhibition, competitive inhibition and behavioural inhibition during the transition between infancy and toddlerhood. https://doi.org/10.31234/OSF.IO/MHKAJ

Holmboe, K., Larkman, C., Klerk, C. de, Simpson, A., Bell, M. A., Patton, L., Christodoulou, C., & Dvergsdal, H. (2021). The Early Childhood Inhibitory Touchscreen Task: A new measure of response inhibition in toddlerhood and across the lifespan. PloS One, 16(12). https://doi.org/https://doi.org/10.1371/journal.pone.0260695

Huppert, T. J., Diamond, S. G., Franceschini, M. A., & Boas, D. A. (2009). HomER: A review of time-series analysis methods for near-infrared spectroscopy of the brain. Applied Optics, 48(10). https://doi.org/10.1364/AO.48.00D280

Jenkinson, M., Pechaud, M., & Smith, S. (2005). BET2-MR-Based Estimation of Brain, Skull and Scalp Surfaces. Human Brain Mapping, 17(2), 143–155. www.fmrib.ox.ac.uk/analysis/research/bet

Kim, H.-Y. (2013). Statistical notes for clinical researchers: assessing normal distribution (2) using skewness and kurtosis. Restorative Dentistry & Endodontics, 38(1), 52. https://doi.org/10.5395/rde.2013.38.1.52

Lloyd-Fox, S., Blasi, A., & Elwell, C. E. (2010). Illuminating the developing brain: The past, present and future of functional near infrared spectroscopy. Neuroscience and Biobehavioral Reviews, 34(3), 269–284. https://doi.org/10.1016/j.neubiorev.2009.07.008

Lui, K. Y. K., Hendry, A., Fiske, A., Dvergsdal, H., & Holmboe, K. (2021). Associations between touchscreen exposure and hot and cool inhibitory control in 10-month-old infants. Infant Behavior and Development, 65, 101649. https://doi.org/10.1016/J.INFBEH.2021.101649

Schweiger, M., & Arridge, S. (2014). The Toast++ software suite for forward and inverse modeling in optical tomography. Journal of Biomedical Optics, 19(4), 040801. https://doi.org/10.1117/1.jbo.19.4.040801

Shi, F., Yap, P.-T., Wu, G., Jia, H., Gilmore, J. H., Lin, W., & Shen, D. (2011). Infant Brain Atlases from Neonates to 1- and 2-Year-Olds. PLoS ONE, 6(4), e18746. https://doi.org/10.1371/journal.pone.0018746

Taga, G., Homae, F., & Watanabe, H. (2007). Effects of source-detector distance of near infrared spectroscopy on the measurement of the cortical hemodynamic response in infants. NeuroImage, 38(3), 452–460. https://doi.org/10.1016/J.NEUROIMAGE.2007.07.050
